# The CuFe_2_O_4_@SiO_2_@ZrO_2_/SO_4_^2−^/Cu nanoparticles: an efficient magnetically recyclable multifunctional Lewis/Brønsted acid nanocatalyst for the ligand- and Pd-free Sonogashira cross-coupling reaction in water†

**DOI:** 10.1039/c9ra03406d

**Published:** 2019-07-03

**Authors:** Mohammad Ali Nasseri, Seyyedeh Ameneh Alavi, Milad Kazemnejadi, Ali Allahresani

**Affiliations:** Department of Chemistry, Faculty of Science, University of Birjand P. O. Box 97175-615 Birjand Iran manaseri@birjand.ac.ir

## Abstract

Herein, the synthesis and application of copper-incorporated sulfated zirconium oxide supported on CuFe_2_O_4_ NPs (CuFe_2_O_4_@SiO_2_@ZrO_2_/SO_4_^2−^/Cu NPs) as a novel Lewis/Brønsted acid nanocatalyst were studied for the Sonogashira C–C cross-coupling reaction. The fabricated CuFe_2_O_4_@SiO_2_@ZrO_2_/SO_4_^2−^/Cu catalyst exhibited efficient activity for a large variety of aryl iodides/bromides and, most importantly, aryl chlorides in water and in the presence of NaOH as a base in short reaction times. The catalyst was fully characterized by FTIR, TG-DTG, VSM, XRD, EDX, FE-SEM and TEM analyses. A synergetic effect could be considered to have arisen from the various Lewis acid and Brønsted acid sites present in the catalyst. The efficient incorporation of copper into zirconia provided a robust highly stable hybrid, which prevented any metal leaching, whether from the magnetite moiety and/or Cu sites in the reaction mixture. Moreover, the catalyst was successfully recovered from the mixture by a simple external magnet and reused for at least 9 consecutive runs. Zero metal leaching, stability, consistency with a variety of substrates, fast performance, cost-effectiveness, environmental friendliness, and preparation with accessible and cheap materials are some of the advantages and highlights of the current protocol.

## Abstract 2

A mild and green protocol was developed by immobilizing copper-incorporated sulfated zirconium oxide on CuFe_2_O_4_ as an efficient inorgano-nanocatalyst for the Sonogashira reaction.

## Graphical abstract

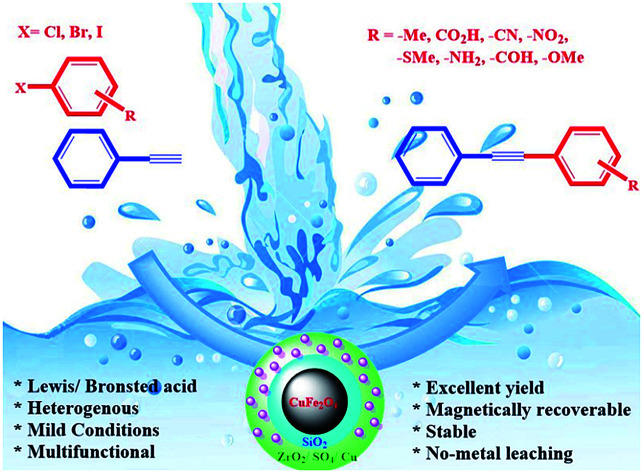

## 1. Introduction

**Scheme 1:**
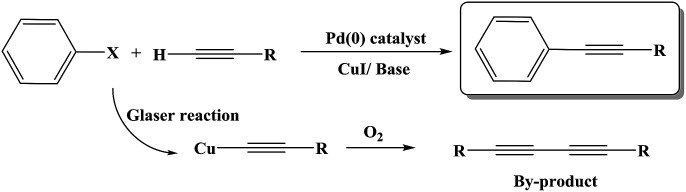
Traditional Sonogashira C–C cross coupling reaction and its possible diyne by-product formation.

The Sonogashira C–C cross-coupling reaction is one of the most applicable types of C–C cross-couplings, which involves the coupling of vinyl or aryl halides or triflates with terminal alkynes (C_sp^2^_–C_sp_);^1,2^ since its vital application for the construction of complex biological and pharmaceutical molecules from simple precursors, the Sonogashira reaction has had significant importance in the field of synthetic organic chemistry.^3^ The reaction was first developed using Pd and Cu as a catalyst and a co-catalyst, respectively (Scheme 1). Since the discovery of this reaction, various methodologies have been developed to resolve its impediments. Palladium is a toxic, rare and expensive transition metal that is mainly used along with toxic and air-sensitive phosphine ligands.^4^ More importantly, the presence of copper as a co-catalyst promotes the Glaser-type homo-coupling of terminal acetylenes to generate a by-product (Scheme 1); thus, various attempts have been developed to perform the reaction under copper-free and Pd-free conditions in a mild, safe, ecofriendly, and cost-effective manner; in this regard, one strategy is the use of cheaper and safer alternative transition metals including Ni,^5^ Cu,^6^ Fe,^7^ and Co.^8^ Among these, the potential of Cu for application in the C–N as well as C–C cross coupling reactions is well-known;^9^ moreover, several achievements have been reported for the Cu-catalyzed Sonogashira reaction; the recent examples include the use of Cu_2_O/RGO,^10^ Cu/Mn bimetallic,^9^ CuI/PPh_3_/K_2_CO_3_,^11^ CuI/K_3_PO_4_/1,4-dioxane,^12^ and Au·CuFe_2_O_4_@silica as catalysts for this reaction.^2^ Recently, Sun and coworkers^13^ have reported the application of a Cu-MOF derived from two-phase Cu/Cu_2_O-rGO as an efficient catalyst for the Sonogashira reaction.

However, various drawbacks, including harsh reaction conditions, long reaction times, use of expensive and toxic materials, lack of selectivity, lack of environmental sustainability, and low reaction yields, especially for aryl chlorides, are still present in most of the reported protocols; therefore, the development of a promising alternative method is required.

Zirconia is one of the most well-known promising solid acids with significant catalytic activity. It is widely used as an efficient acid catalyst in oil refineries and petrochemical industries for processes such as hydrocarbon conversion, alkylation, cracking, Friedel–Crafts acylation, esterification and isomerization;^14,15^ moreover, the activity of zirconia can be largely promoted by its treatment with sulfate groups, and as a result, sulfated zirconium oxide (ZrO_2_/SO_4_^2−^) is obtained. The high thermal stability, outstanding catalytic activity, high acidity, stability in various organic solvents, and durability under harsh reaction conditions are some of the notable and applicable properties of sulfated zirconia that make it a suitable support for more modifications (an objective of this study) and/or catalytic aspects.^16^ Various catalytic activities, such as towards benzylation,^16^ multicomponent reactions,^17^ and synthesis of dioxane,^18^ of ZrO_2_/SO_4_^2−^ have been reported in the literature; in addition, heterogeneous solid supports can be magnetized by magnetic nanoparticles (MNPs) to make these supports magnetically recoverable;^19^ moreover, due to their high aspect ratio, MNPs can strongly improve the catalytic activity of a catalyst.^3^

In this study, we introduced copper-incorporated sulfated zirconium oxide supported on CuFe_2_O_4_ nanoparticles as an efficient, recyclable and durable magnetic nanocatalyst for the first time for the C–C cross coupling reaction of phenylacetylene with aryl iodides, aryl bromides and aryl chlorides under mild reaction conditions. The present system not only benefits from the durable ZrO_2_/SO_4_^2−^ solid support, but also the magnetic CuFe_2_O_4_ magnetic core in the catalyst provides suitable recyclability to the catalyst *via* an external magnet.

## 2. Experimental

### 2.1. Instrumentation and materials

All chemicals were freshly purchased from Sigma and Merck or Fluka Chemical Companies with no further purification. All solvents were distilled under a N_2_ atmosphere and dried before use. The reaction progress was monitored by thin layer chromatography (TLC). The FTIR spectra were obtained *via* the JASCO FT/IR 4600 spectrophotometer using KBr pellets. The ^1^H NMR (250 MHz) and ^13^C NMR (62.9 MHz) spectroscopies were performed by the Bruker Avance DPX-250 spectrometer in CDCl_3_ and DMSO-*d*_6_ as solvents, respectively. TMS was used as an internal standard. Mass spectrometry was performed using the Thermolyne 79300 model tube furnace equipped with the MKS gas analyzer coupled to a quadrupole mass selective detector. The scanning electron microscopy images (FE-SEM) were obtained by the TESCAN MIRA3 apparatus. Transmission electron microscopy (TEM) was conducted using the Philips EM208 microscope at 100 kV. The magnetic behavior of the samples was investigated using the Lake Shore Cryotronics 7407 vibrating sample magnetometer (VSM) at room temperature. EDX spectroscopy was performed using a field-emission scanning electron microscope (FESEM, JEOL 7600F), equipped with an X-ray energy dispersive spectrometer obtained from Oxford instruments. The TGA of the samples was performed using NETZSCH STA 409 PC/PG under a N_2_ atmosphere at the heating rate of 10 °C min^−1^ in the temperature range of 25–850 °C. Metal leaching studies were performed using the VARIAN VISTA-PRO CCD simultaneous ICP-OES instrument as well as the inductively coupled plasma mass spectrometer (ICP-MS) Thermo Elemental VG PQ ExCell.

### 2.2. Preparation of the CuFe_2_O_4_@SiO_2_ MNPs

The CuFe_2_O_4_ NPs were prepared according to a previously reported procedure.^20^ Cu(NO_3_)_2_ (10 mmol, 1.9 g) and Fe(NO_3_)_3_ (20 mmol, 4.8 g) were dissolved in water (75 mL) and ultrasonicated for 30 min. Then, NaOH (25 mL, 1 N) was added dropwise to the abovementioned solution until a reddish-black sediment was formed. The reaction mixture was stirred at 90 °C for 2 h. The reddish-black precipitate was washed with water (2 × 25 mL) and EtOH (2 × 25 mL) until the pH of the solution was adjusted to 7.0. The sediment was separated, dried in a vacuum oven for 12 h, and then calcined in a furnace at 700 °C for 5 h at the heating rate of 20 °C min^−1^. The CuFe_2_O_4_@SiO_2_ MNPs were synthesized using the sol–gel method. CuFe_2_O_4_ (2.0 g, 8.5 mmol) was ultrasonically dispersed in ethanol (25 mL) for 2 h at 60 °C, and then, aqueous NaOH (10% w/w, 10 mL) was added to the mixture followed by stirring at room temperature for 30 min. Then, tetraethoxyorthosilicate (TEOS, 1.0 mL) was added to the mixture, and stirring was continued for further 24 h. The CuFe_2_O_4_@SiO_2_ MNPs were separated from the solution by an external magnetic field, washed with water (3 × 5 mL) and EtOH (2 × 5 mL), and then dried under vacuum for 48 h. The resultant CuFe_2_O_4_@SiO_2_ MNPs were calcined at 800 °C for 4 h at the heating rate of 20 °C min^−1^.

### 2.3. Preparation of the CuFe_2_O_4_@ZrO_2_/SO_4_^2−^/Cu NPs

**Scheme 2:**
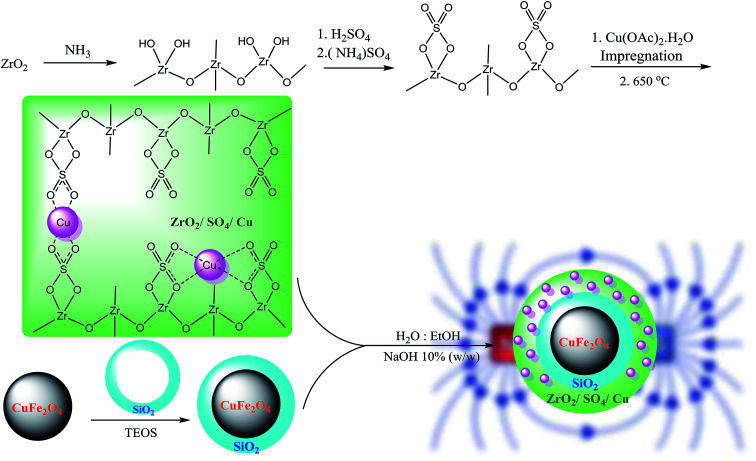
Synthesis of CuFe_2_O_4_@SiO_2_@ZrO_2_/SO_4_^2−^/Cu nanoparticles.

Sulfated zirconium oxide was prepared according to a previously reported protocol with slight modification.^21,22^ At first, ZrCl_4_ (2.3 g, 10 mmol) as a precursor was dissolved in 10 mL deionized water. The ammonia solution (10 mL, 1 N) was added dropwise for 30 min until the pH was adjusted to 11. The resulting suspension was aged for 24 h at room temperature. Then, the obtained white sediment was washed with deionized water using centrifugation (5 × 10 mL) until the solution was neutralized (pH = 7). The resultant Zr(OH)_4_ was dried at 100 °C for 24 h. The isolated yield for Zr(OH)_4_ was found to be 96% (1.5 g). Subsequently, Zr(OH)_4_ (1.0 g) was immersed in an (NH_4_)_2_SO_4_ solution (2.5 g in 50 mL distilled water) with the mixing ratio of 1 : 3, Zr : S. The solution was dried at 100 °C for 24 h followed by calcination at 650 °C for 3 h in a furnace at the heating rate of 10 °C min^−1^. The resultant ZrO_2_/SO_4_^2−^ (1.0 g) was impregnated in the Cu(OAc)_2_ solution (0.1 g in 50 mL distilled water) to obtain the Cu content of 10 wt% (theoretically). The mixture was dried at 100 °C for 12 h and subsequently calcined at 400 °C for 3 h under an air atmosphere at the heating rate of 10 °C min^−1^. The obtained ZrO_2_/SO_4_^2−^/Cu was washed with deionized water (3 × 10 mL) and then dried in an oven (50 °C) for 8 h. The prepared ZrO_2_/SO_4_^2−^/Cu NPs were supported on CuFe_2_O_4_@SiO_2_ magnetic nanoparticles. A mixture of CuFe_2_O_4_@SiO_2_ NPs (0.2 g) in H_2_O : EtOH (15 mL, 1 : 3 v/v) was sonicated for 10 min at room temperature. A pre-dispersed ethanolic solution of ZrO_2_/SO_4_^2−^ (0.4 g) was added to the abovementioned mixture followed by sonication for additional 10 min at room temperature. NaOH 10% w/w (15 mL) was added dropwise to the solution for 30 min under sonication. The solution was stirred for 24 h. The resultant CuFe_2_O_4_@ZrO_2_/SO_4_^2−^/Cu NPs were separated by an external magnetic, washed with deionized water and dried in an oven for 12 h. The complete route for the preparation of the catalyst is shown in Scheme 2.

### 2.4. General procedure for the Sonogashira coupling reaction using the CuFe_2_O_4_@SiO_2_@ZrO_2_/SO_4_^2−^/Cu nanoparticles

A mixture of phenylacetylene (1.5 mmol), aryl halide (1.0 mmol), the CuFe_2_O_4_@SiO_2_@ZrO_2_/SO_4_^2−^/Cu magnetic nanocatalyst (0.005 g, 0.3 mol% Cu), NaOH (1.0 mmol) and water (1.0 mL) was stirred at 60 °C in an oil bath. The reaction progress was monitored by TLC. Upon completion of the reaction, the catalyst was magnetically removed, and the mixture was extracted with 10 mL of Et_2_O. The combined organic layers were dried over MgSO_4_, and then, the solvent was removed under reduced pressure. The desired pure coupling product was obtained by flash chromatography of the crude product.

## 3. Results and discussion

### 3.1. Catalyst characterization

**Fig. 1:**
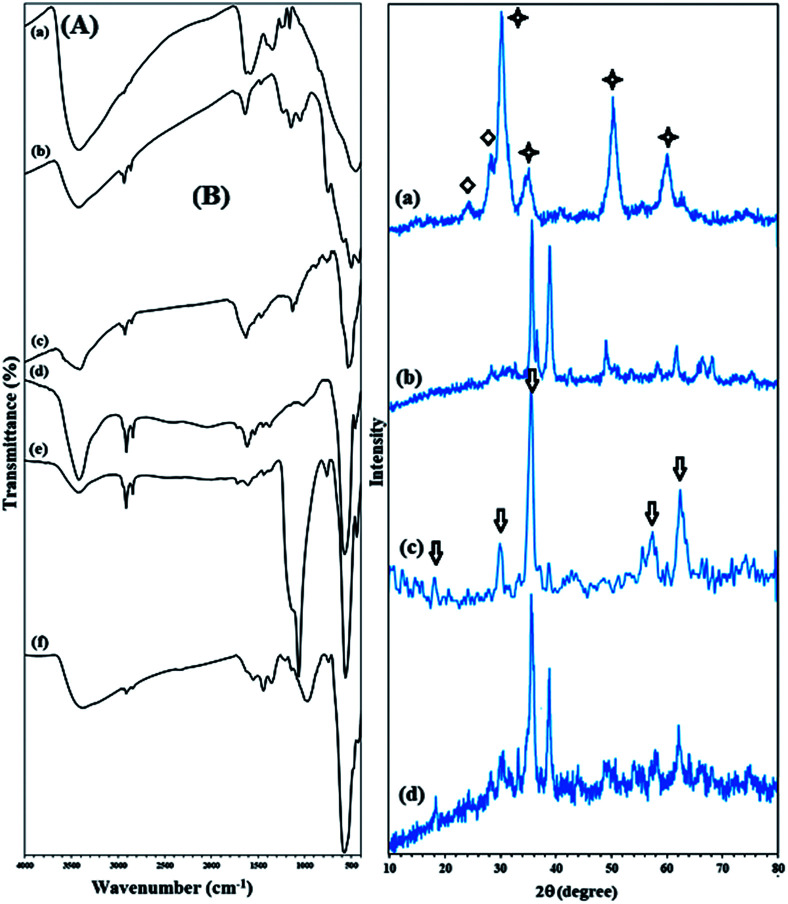
(A) FTIR spectra of (a) Zr(OH)_4_, (b) ZrO_2_/SO_4_^2−^, (c) ZrO_2_/SO_4_^2−^/Cu, (d) CuFe_2_O_4_, (e) CuFe_2_O_4_@SiO_2_, and (f) CuFe_2_O_4_@SiO_2_@ZrO_2_/SO_4_^2−^/Cu. (B) XRD pattern of (a) ZrO_2_/SO_4_^2−^, (b) ZrO_2_/SO_4_^2−^/Cu, (c) CuFe_2_O_4_, and (d) CuFe_2_O_4_@SiO_2_@ZrO_2_/SO_4_^2−^/Cu. The star indicates a tetragonal structure, and the diamond represents a monoclinic structure.

The FTIR spectra of Zr(OH)_4_, ZrO_2_/SO_4_^2−^, ZrO_2_/SO_4_^2−^/Cu, and CuFe_2_O_4_@SiO_2_@ZrO_2_/SO_4_^2−^/Cu are shown in Fig. 1A(a). The broad and resolved peak at 3403 cm^−1^ related to the O–H stretching vibration confirmed the hydration of zirconium chloride using ammonia. Moreover, the absorption peaks related to Zr–O–Zr could be seen at 640–750 cm^−1^ (Fig. 1A(a)). The FTIR spectrum of ZrO_2_/SO_4_^2−^ demonstrated characteristic peaks at 1143, 1044, and 994 cm^−1^ (as a shoulder), which corresponded to the asymmetric or symmetric stretching vibrations of the S=O or S–O bonds (Fig. 1A(b)).^23^ These vibrations were characteristic for the bidentate sulfate ions coordinated to a metal cation. The series of peaks at 467–747 cm^−1^ were assigned to the Zr–O–Zr asymmetric stretching vibrations.^24^ Moreover, the broad peak at 3421 cm^−1^ and the medium peak at 1636 cm^−1^ were assigned to the O–H stretching and bending vibrations of the adsorbed and/or coordinated water by the sulfate groups, respectively.^25^

A sharp peak near 500 cm^−1^ was attributed to the Cu–O stretching vibration, demonstrating that the incorporation of the Cu ions took place through the oxygen atoms of the sulfated ions in ZrO_2_/SO_4_^2−^/Cu; moreover, this subsequently confirmed the successful coordination of the Cu cations to the catalyst framework (Fig. 1A(c)).^26^ The stretching vibrations related to Zr–O–Zr were covered due to this strong absorption.

In the CuFe_2_O_4_ FTIR spectrum, two absorptions at 1629 and 3435 cm^−1^ represented the H–O–H bending and free O–H stretching vibrations, respectively, due to the water molecules adsorbed on the surface of the CuFe_2_O_4_ NPs with high aspect ratio.^27^ The two absorption bands at 476 and 590 cm^−1^ were assigned to the Cu–O and Fe–O stretching vibrations, respectively (Fig. 1A(d)).^27,28^ The strong absorption at 1093 cm^−1^ (Si–O vibrations) confirmed the successful coating of the CuFe_2_O_4_ NPs with a silica shell (Fig. 1A(e)).

The presence of vibration bands at 421, 575, and 870–1148 cm^−1^, which were due to Fe–O, Cu–O, and Si–O–Si, respectively, demonstrated that ZrO_2_/SO_4_^2−^/Cu was successfully supported on CuFe_2_O_4_@SiO_2_ (Fig. 1A(f)). In addition, the presence of several bands with medium intensity in the 1361–1641 cm^−1^ region was allocated to the ZrO_2_/SO_4_^2−^ stretching vibrations (Fig. 1A(f)).

The XRD patterns of ZrO_2_/SO_4_^2−^, ZrO_2_/SO_4_^2−^/Cu, and CuFe_2_O_4_@SiO_2_@ZrO_2_/SO_4_^2−^/Cu are shown in Fig. 1B. ZrO_2_/SO_4_^2−^ demonstrated three characteristic peaks with strong intensities at 2*θ* = 30.4°, 50.3° and 60.2°, which represented the tetragonal structure of ZrO_2_/SO_4_^2−^ with high crystallinity (JCPDS 17-0923) (Fig. 1B(a)).^21,29^ More precisely, a mixture of the monoclinic and tetragonal phases was observed in the spectrum (Fig. 1B(a), stars and diamonds represent a tetragonal and monoclinic structure, respectively) that was in agreement with the reported ZrO_2_/SO_4_^2^ crystal structure.^21,29^ The peaks with lower intensities at 2*θ* = 24.1° and 28.3° were assigned to the monoclinic structure of zirconia.

Note that the presence of sulfated groups does not lead to a phase change of zirconia; this is may be due to strong interactions between zirconia and the sulfate ions.^29^ On the other hand, based on the presence of prominent peaks related to the trigonal structure of ZrO_2_/SO_4_^2−^, it could be concluded that the impregnation of the sulfate ions showed a strong effect on the phase modification of zirconia from a thermodynamically more stable monoclinic phase to a metastable tetragonal phase. The incorporation of Cu into the structure of ZrO_2_/SO_4_^2−^ caused a small shift of the peaks related to the sulfated zirconia crystal structure at 2*θ* = 31.7°, 35.6°, 38.8°, 48.4°, 58.2°, 61.8°, 66.0°, and 68.08°. However, the peaks at 2*θ* = 35.6°, 38.8°, 48.4°, 66.0°, and 68.08° matched well with the indices (002), (111), (202), (311), and (113), respectively, which corresponded to the thermally prepared CuO powder structure (Fig. 1B(b)).^30,31^ Furthermore, the peaks with much lower intensities near to baseline indicating the crystal structure of the sulfated zirconium oxide (Fig. 1B(b)). The results suggested that the Cu–O bond between the sulfate groups in ZrO_2_/SO_4_^2−^/Cu and the Cu ions was completely in agreement with its corresponding FTIR spectrum. The XRD pattern of the CuFe_2_O_4_ NPs represented Bragg's reflections at 2*θ* = 18.6°, 30.2°, 35.5°, 57.8°, and 62.80° (arrows in Fig. 1B(c)) corresponding to their indices (101), (200), (211), (321) and (400). These reflections were consistent with the tetragonal crystal structure of CuFe_2_O_4_ (JCPDS card no. 34-0425), in agreement with those reported in the literature (Fig. 1B(c)).^2,27,28^Fig. 1B(d) shows the crystal structure of the catalyst CuFe_2_O_4_@SiO_2_@ZrO_2_/SO_4_^2−^/Cu. The peak intensities for the CuFe_2_O_4_ crystal structure were reduced when ZrO_2_/SO_4_^2−^ was supported on the CuFe_2_O_4_@SiO_2_ surface. The results confirmed that the functionalization of CuFe_2_O_4_ did not lead to a phase change of ZrO_2_/SO_4_^2^. Moreover, the presence of a sharp peak at 2*θ* = 38.2° demonstrated the incorporation of Cu into the catalyst. The peaks at 2*θ* = 60.0°, 50.3°, 30.2°, and 50.7° may be assigned to the ZrO_2_/SO_4_^2−^ crystal structure.

**Fig. 2:**
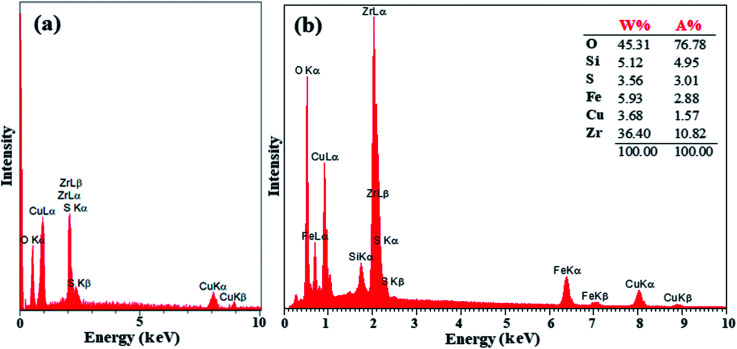
EDX spectra of (a) ZrO_2_/SO_4_^2^/Cu and (b) CuFe_2_O_4_@SiO_2_@ZrO_2_/SO_4_^2−^/Cu.

**Fig. 3:**
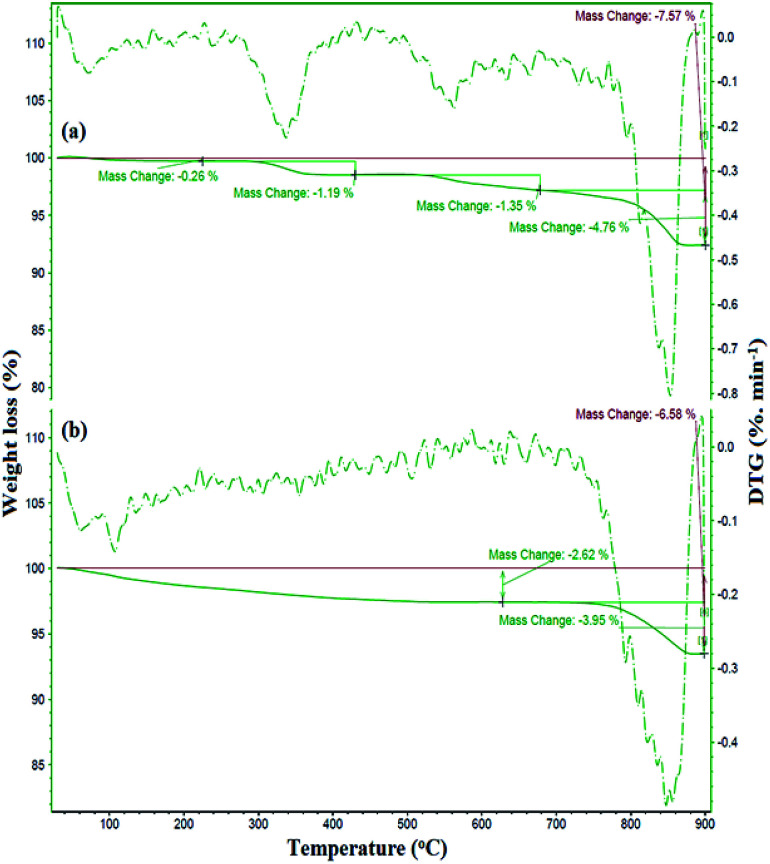
TGA-DTG curves of (a) ZrO_2_/SO_4_^2−^/Cu and (b) CuFe_2_O_4_@SiO_2_@ZrO_2_/SO_4_^2−^/Cu.

The preparation of ZrO_2_/SO_4_^2−^ was confirmed by the presence of the Cu, Zr, O, and S elements, which were detected by EDX analysis (Fig. 2a). Moreover, the presence of these elements in the catalyst was investigated and confirmed by EDX analysis. As shown in Fig. 2b, the elements Zr, Cu, O, S, Fe, and Si were detected in the catalyst. The thermal behavior of ZrO_2_/SO_4_^2−^ and the catalyst is shown in Fig. 3a. ZrO_2_/SO_4_^2−^ showed a significant thermal stability, and only a 7.5% weight loss was observed in the temperature range of 25–1000 °C (Fig. 3a). This degradation occurred in four steps, where the first and second steps were assigned to the loss of the physically adsorbed water from the catalyst surface (0.26% weight loss at 210 °C), and the escape of the trapped water in the catalyst network by sulfate groups (1.19% weight loss at 350 °C), respectively. The third weight loss in the temperature range of 530–665 °C was related to the oxidation of copper and the formation of CuO.^32^ The weight loss that appeared in the temperature range of 680–860 °C was due to the decomposition of sulfate as well as structural OH^−^ groups.^33–35^ The decoration of ZrO_2_/SO_4_^2−^/Cu on CuFe_2_O_4_@SiO_2_ improved the thermal stability of the catalyst, and only 6.5% weight loss was observed until 1000 °C. The first weight loss with a mild slope, which lasted till 780 °C, was due to the loss of the adsorbed water in the crystal structure of the catalyst. The subsequent weight loss was attributed to the decomposition of incorporated Cu and sulfate groups with a 4.0% weight loss (Fig. 3b).

**Fig. 4:**
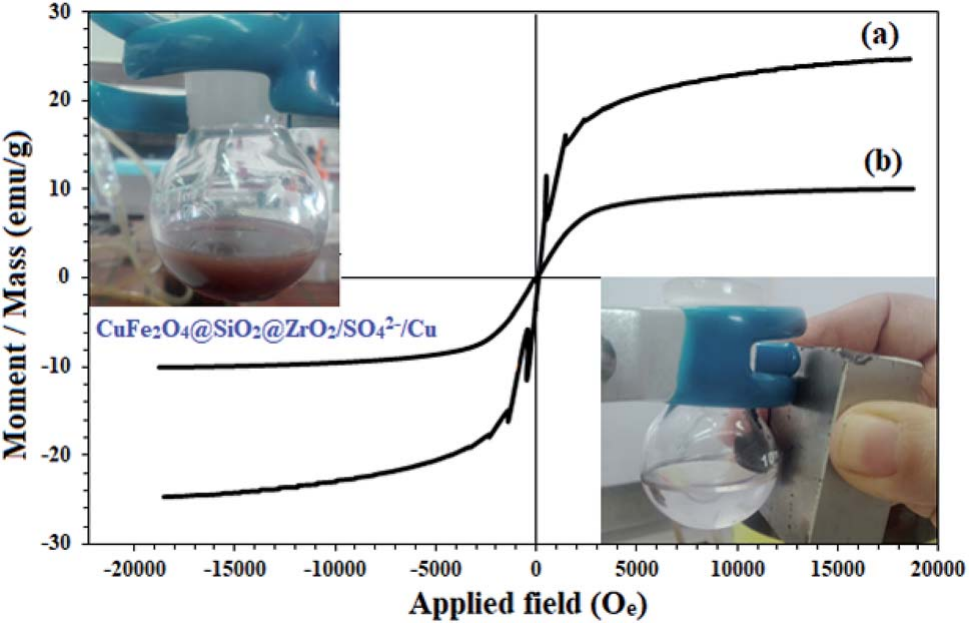
Magnetic behavior of (a) CuFe_2_O_4_ and (b) CuFe_2_O_4_@SiO_2_@ZrO_2_/SO_4_^2−^/Cu.

The magnetic properties of CuFe_2_O_4_ and CuFe_2_O_4_@SiO_2_@ZrO_2_/SO_4_^2−^/Cu were studied by VSM analysis (Fig. 4). As shown in Fig. 4, the samples represented a superparamagnetic behavior with no hysteresis loops in their spectra. The saturation magnetization for CuFe_2_O_4_ was found to be 24.6 emu g^−1^ (Fig. 4a). This amount was largely reduced to 10.1 emu g^−1^ for CuFe_2_O_4_@SiO_2_@ZrO_2_/SO_4_^2−^/Cu; this strongly confirmed its surface functionalization (Fig. 4b). However, there was sufficient magnetic response for the complete separation of nanoparticles from the mixture. The inset figures show the immediate separation of CuFe_2_O_4_@SiO_2_@ZrO_2_/SO_4_^2−^/Cu from the mixture in 120 seconds under an applied external magnetic field after the dispersion of CuFe_2_O_4_@SiO_2_@ZrO_2_/SO_4_^2−^/Cu.

**Fig. 5:**
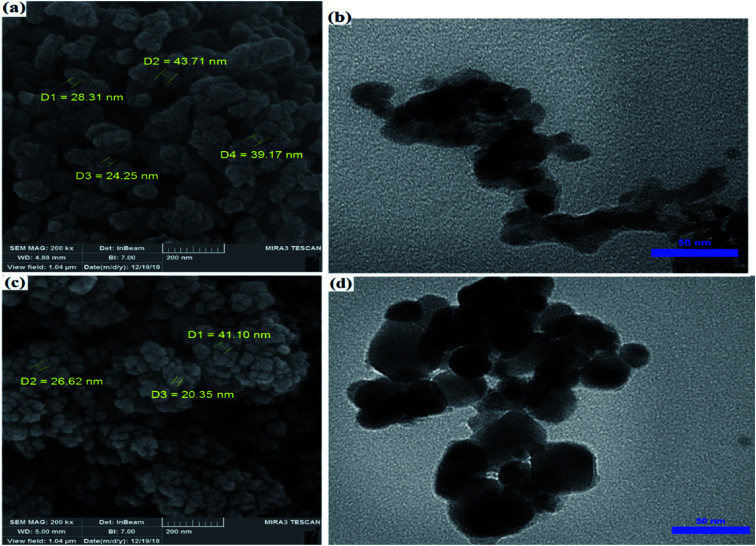
(a) FE-SEM and (b) TEM images of ZrO_2_/SO_4_^2−^/Cu. (c) FE-SEM and (d) TEM images of CuFe_2_O_4_@SiO_2_@ZrO_2_/SO_4_^2−^/Cu.

The morphology and shape of ZrO_2_/SO_4_^2−^/Cu and CuFe_2_O_4_@SiO_2_@ZrO_2_/SO_4_^2−^/Cu were studied by the SEM and TEM techniques. The SEM images of ZrO_2_/SO_4_^2−^/Cu and CuFe_2_O_4_@SiO_2_@ZrO_2_/SO_4_^2−^/Cu represented an irregularly shaped amorphous morphology that could be due to the expected agglomeration of activated ZrO_2_/SO_4_^2−^ by the inclusion of transition metals (Fig. 5a and b).^36^ By comparing their TEM images (Fig. 5c and d), this agglomeration was more clearly observed. According to Fig. 5c and d, the particles had the average size of 15 nm and 40 nm for ZrO_2_/SO_4_^2−^/Cu and CuFe_2_O_4_@SiO_2_@ZrO_2_/SO_4_^2−^/Cu, respectively.

### 3.2. Optimization of reaction parameters

To find premium reaction conditions for the Sonogashira C–C coupling reaction, the reaction of iodobenzene with phenylacetylene was chosen as the model reaction. The effects of the reaction parameters, such as the type of base, reaction temperature, solvent, and catalyst amount, were studied. The results are presented in Table 1. The reaction obviously proceeded in polar-protic solvents such as EtOH, MeOH and water (Table 1, entries 1, 8, and 12). These results were in agreement with the structure of the catalyst containing hydrophilic groups as well as the mechanism proposed in the next section. Other solvents provided low to moderate yields. The highest efficiency was obtained in water after reaction for 30 min in the presence of 0.005 g of catalyst (entry 12, 92%). There was no satisfactory conversion under solvent-free conditions (Table 1, entry 11). NaOH and KOH were found to be efficient bases for this transformation (Table 1, entries 12 and 13). Moreover, 60 °C and 0.005 g of the catalyst were the premium temperature and catalyst amount for the model reaction, respectively (Table 1, entries 12, 21–26).

**Table 1:** Optimization of the reaction parameters for the reaction of phenylacetylene with iodobenzene catalyzed by CuFe_2_O_4_@SiO_2_@ZrO_2_/SO_4_^2−^/Cu

Entry	Solvent	Base	Catalyst (g)	*T* (°C)	*t* (min)	Yield (%)
1	EtOH	NaOH	0.005	60	30	80
2	CH_3_CN	NaOH	0.005	60	30	69
3	THF	NaOH	0.005	60	30	40
4	CH_2_Cl_2_	NaOH	0.005	60	30	50
5	Toluene	NaOH	0.005	60	30	75
6	CHCl_3_	NaOH	0.005	60	30	Trace
7	DMSO	NaOH	0.005	60	30	89
8	MeOH	NaOH	0.005	60	30	88
9	Dioxane	NaOH	0.005	60	30	45
10	DMF	NaOH	0.005	60	30	75
11	Solvent-free	NaOH	0.005	60	30	Trace
**12**	**H**_**2**_ **O**	**NaOH**	**0.005**	**60**	**30**	**92**
13	H_2_O	KOH	0.005	60	30	92
14	H_2_O	K_2_CO_3_	0.005	60	30	66
15	H_2_O	K_3_PO_4_	0.005	60	30	90
16	H_2_O	NaOAC	0.005	60	30	90
17	H_2_O	LiHMDS	0.005	60	30	55
18	H_2_O	Et_3_N	0.005	60	30	76
19	H_2_O	HMTA	0.005	60	30	54
20	H_2_O	*t*-BuOK	0.005	60	30	88
21	H_2_O	NaOH	0.001	60	30	77
22	H_2_O	NaOH	0.003	60	30	82
23	H_2_O	NaOH	0.01	60	30	90
24	H_2_O	NaOH	0.005	R.T.	30	88
25	H_2_O	NaOH	0.005	80	30	92
26	H_2_O	NaOH	0.005	Ref.	30	93

The scope of the reaction was investigated and extended with a variety of aryl halides and phenylacetylene in the presence of CuFe_2_O_4_@SiO_2_@ZrO_2_/SO_4_^2−^/Cu NPs as the catalyst under the previously obtained optimum conditions. As shown in Table 2, the method tolerated various substrates bearing either electron-donating and/or electron-withdrawing substituents, and high-to-excellent yields were obtained for all substrates (Table 2). Generally, the substrates with electron-withdrawing substituents provide higher efficiencies than others in terms of time and yield (Table 2, for example entries 5, 7, and 13). Moreover, iodide as a leaving group accelerated the reaction than Br or Cl. The results are in agreement with an oxidative addition/reductive elimination mechanism, which has been discussed hereinafter.

**Table 2:** Sonogashira cross-coupling reaction of phenylacetylene with various aryl halides catalyzed by the CuFe_2_O_4_@SiO_2_@ZrO_2_/SO_4_^2−^/Cu NPs

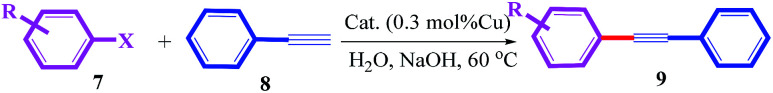							
Entry	R	X	9	Time (min)	Yield^a^ (%)	TON	TOF (h^−1^)
1	H	I	9a	30	92	326	562
2	H	Cl	9b	100	88	293	177
3	H	Br	9c	45	92	307	409
4	4-Me	I	9d	30	94	313	626
5	4-CO_2_H	I	9e	20	89	297	873
6	4-NH_2_	Br	9f	55	76	253	275
7	2-NO_2_	I	9g	20	90	300	909
8	3-Me	I	9h	60	82	273	273
9	2-NH_2_	Cl	9i	150	69	230	92
10	4-CO_2_H	Br	9j	70	78	260	224
11	4-SMe	Br	9k	90	58	193	128
12	3-NH_2_	Cl	9l	180	70	233	77
13	4-CN	Br	9m	25	92	307	730
14	4-COH	I	9n	65	88	293	271
15	4-OMe	I	9o	55	88	293	318
16	3-SMe	Cl	9p	110	68	227	124
17	4-NO_2_	I	9q	30	98	326	196
18	2-SMe	Cl	9r	140	72	240	103
19	4-NO_2_	Br	9s	45	97	323	430

a Reaction conditions: aryl halide (1.0 mmol), phenylacetylene (1.5 mmol), NaOH (1.0 mmol), H_2_O (2 mL), catalyst (0.3 mol% Cu), and 60 °C.

### 3.3. Mechanistic study

At first, we investigated the model reactions in the presence of CuFe_2_O_4_, CuFe_2_O_4_@SiO_2_, ZrCl_4_, ZrO_2_/SO_4_^2−^, and ZrO_2_/SO_4_^2−^/Cu as control experiments. The corresponding results are summarized in Table 3. As shown in the Table, ZrCl_4_, (NH_4_)_2_SO_4_, ZrO_2_/SO_4_^2−^, and Cu(OAc)_2_ did not afford any coupling products under the reaction conditions. Interestingly, the CuFe_2_O_4_ NPs demonstrated catalytic activity for the reaction (Table 3, entry 6). This activity was reduced after coating of these NPs with a silica shell; this was further evidence for the catalytic activity of CuFe_2_O_4_ NPs (Table 3, entries 6 and 7). Previously, Gholinejad and coworkers^2^ have reported a possible interference of CuFe_2_O_4_@silica as a catalyst towards the Sonogashira coupling reaction; this is in agreement with the results obtained from the control experiments. ZrO_2_/SO_4_^2−/^Cu provided a 76% isolated yield (Table 3, entry 8). The results suggested that (i) the catalytic activity of CuFe_2_O_4_@SiO_2_/ZrO_2_/SO_4_^2−^/Cu originated due to the incorporation of copper into the catalyst, and (ii) a synergetic effect could be considered to have originated from the various functionalities of the catalyst that promoted the coupling reaction; accordingly, the yield obtained using CuFe_2_O_4_@SiO_2_/ZrO_2_/SO_4_^2−^/Cu is roughly the sum of the yields obtained using CuFe_2_O_4_@SiO_2_ and ZrO_2_/SO_4_^2−^/Cu separately (Table 3, entries 7 and 8); this finding suggests that both parts of the final catalyst contribute to the catalytic activity.

**Table 3:** Control experiments for the reaction of phenylacetylene with iodobenzene catalyzed by CuFe_2_O_4_@SiO_2_/ZrO_2_/SO_4_^2−^/Cu

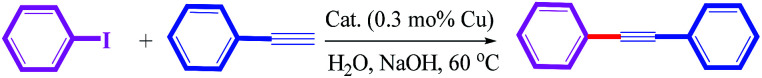		
Entry	Catalyst	Yield (%)
1	No catalyst	No reaction
2	ZrCl_4_	No reaction
3	(NH_4_)_2_SO_4_	No reaction
4	ZrO_2_/SO_4_^2-^	No reaction
5	Cu(OAC)_2_	No reaction
6	CuFe_2_O_4_	26%
7	CuFe_2_O_4_@SiO_2_	15%
8	ZrO_2_/SO_4_^2−^/Cu	76%
9	CuFe_2_O_4_@SiO_2_/ZrO_2_/SO_4_^2−^/Cu	92%

**Scheme 3:**
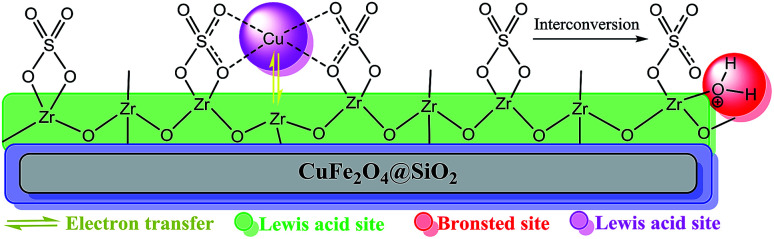
The most possible structure and various acid functionalities of CuFe_2_O_4_@SiO_2_@ZrO_2_/SO_4_^2−^/Cu.

**Scheme 4:**
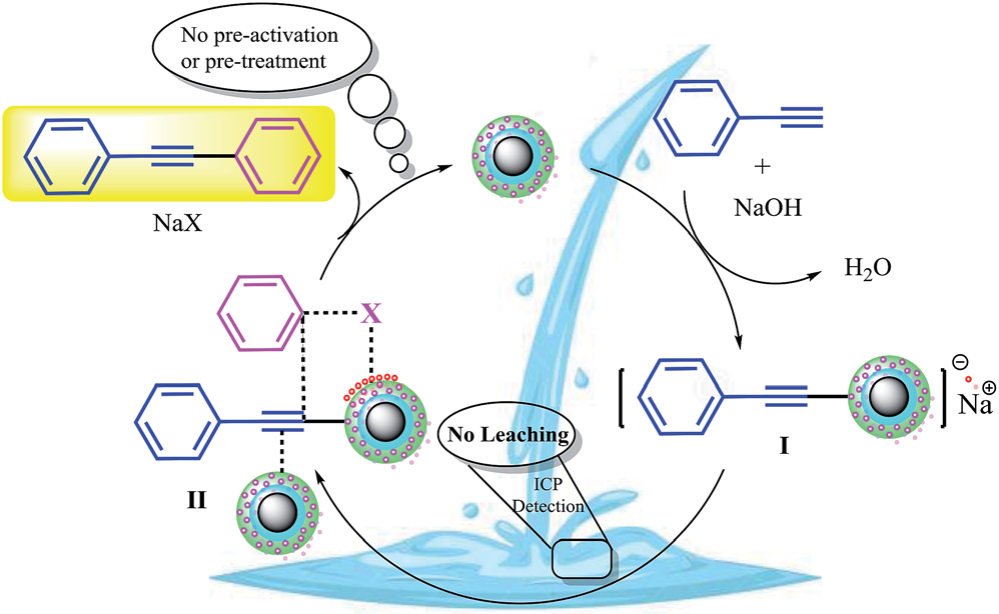
A tentative mechanism for the Sonogashira cross-coupling reactions catalyzed by CuFe_2_O_4_@SiO_2_@ZrO_2_/SO_4_^2−^/Cu.

Based on the abovementioned observations, we suggested the most possible mechanistic pathway for this method. According to the results obtained from the control experiments as well as the literature,^10–12,37^ there were several catalytic active sites on the surface of CuFe_2_O_4_@SiO_2_, ZrCl_4_, and ZrO_2_/SO_4_^2−^. Scheme 3 shows a plausible structure for CuFe_2_O_4_@SiO_2_, ZrCl_4_, and ZrO_2_/SO_4_^2−^. The coordinated Cu and zirconium were efficient active Lewis sites. Moreover, water was coordinated through an interconversion reaction between free sulfate groups on the catalyst, and this provided active Brønsted acid sites. The presence of water as a solvent promoted the active Brønsted acid sites (Scheme 3); this explained the high catalytic activity of the catalyst with water as a solvent. Due to the presence of these catalytic active sites in CuFe_2_O_4_@SiO_2_, ZrCl_4_, and ZrO_2_/SO_4_^2−^, a synergetic effect could be speculated for this catalyst, arising from the Cu sites, Zr sites, sulfate groups,^38^ coordinated water,^21^ and CuFe_2_O_4_.^2^ A plausible structure for the catalyst is shown in Scheme 4, which is in agreement with the characterization data as well as the structure proposed in literature.^2,21,38^ In the first step of the proposed mechanism, Cu-acetylide (Scheme 4, intermediate I) was formed *via* oxidative addition with the participation of a base. This addition could be mediated by electron transfer from zirconium to copper (from Cu i to Cu ii for example, see Scheme 3). To prove this claim, the Sonogashira reaction was performed in the presence of CuSO_4_ under the same reaction conditions. No coupling products were found in the mixture. However, it could be concluded that the presence of zirconium in the catalyst was mandatory for electron transfer. A water molecule was formed during this transformation. The hydrophilic nature of the catalyst surface arising from the sulfate groups increased the solubility of the base. Due to interconversion between sulfate groups (Schemes 3 and 4), the cation of the base (Na^+^ in this case) could be coordinated to Zr, and the reaction was accelerated. This step was supported by the Cu-catalyzed Sonogashira reactions^9,11,12,37^ as well as our observations in the control experiments. In the next step, another catalyst molecule formed the π-complex intermediate II, which generated a positive charge in acetylene and thus favored a nucleophilic attack on the electron-rich aryl halide. Due to the presence of the coordinated cation on the catalyst surface (Na^+^ in this case), a four-membered intermediate II, as shown in Scheme 4, was generated. The intermediate II underwent reductive elimination and led to the formation of a C–C coupling product as well as a NaX salt. The catalyst was regenerated for the next cycle (Scheme 4).

**Scheme 5:**
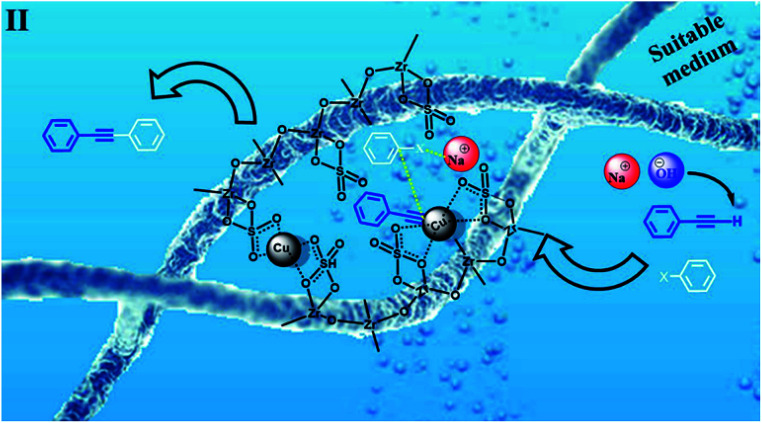
Intermediate II in Scheme 3. A proposed scheme for the possible interaction of the materials in the C–C Sonogashira coupling in water in the presence of CuFe_2_O_4_@SiO_2_@ZrO_2_/SO_4_^2−^/Cu.

According to the proposed catalyst structure shown in Scheme 4, the catalyst can provide a suitable medium for conducting the reaction in water. As shown in Scheme 5, with respect to the nanocomposite structure of the catalysts, organic compounds were introduced into the catalyst by removing water, which contained catalytic active sites including copper and zirconium; as the concentration and number of effective collisions increased, the reaction proceeded with high efficiency. This structure not only addressed the concerns about the transfer of mass in an aqueous medium, but was also consistent with the high efficiencies achieved for the Sonogashira compounds in this study. In the end, the desired product was removed from the medium. This rigid intermediate also prevented the formation of diyne by-products, which were produced by the coupling of two equivalents of Cu-acetylide in the presence of molecular oxygen (Scheme 1, Glaser type reaction).^6^

### 3.4. Recoverability studies

**Fig. 6:**
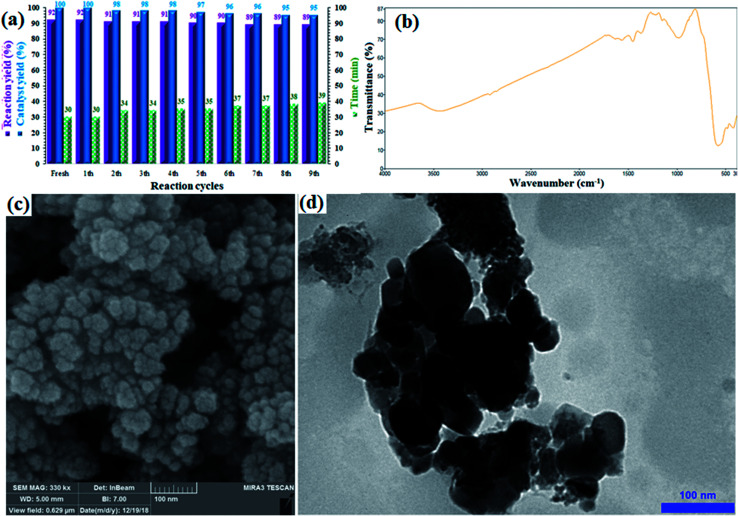
(a) Recovery and reusability of CuFe_2_O_4_@SiO_2_@ZrO_2_/SO_4_^2−^/Cu in the Sonogashira reaction of iodobenzene with phenylacetylene under optimized reaction conditions. (b) FTIR spectrum, (c) FE-SEM, and (d) TEM images of the recovered catalyst after the 9^th^ run.

Stability, durability and, consequently, recyclability of a heterogeneous catalyst are prominent and important factors from economical, energy saving, and environmental points of view;^39–41^ the rigid inorganic structure of the sulfated zirconium oxide solid support along with the magnetic properties of the CuFe_2_O_4_ moieties made the catalyst recoverable and reusable and minimized any metal leaching. The recyclability of the catalyst was investigated in the Sonogashira cross-coupling reaction of phenylacetylene and iodobenzene in the presence of NaOH at 60 °C. The catalyst was recovered in each cycle, washed with EtOH (2 × 5 mL) and reused in the next run without any purification or pre-activation. Fig. 6a shows the corresponding results for nine consecutive runs, and an insignificant loss in efficiency (catalyst yield and reaction yield) was observed.

The yield of the coupling product reached 89% (3% loss) after the 9^th^ run. There was also a very intransigent increase in the reaction time until the 9^th^ cycle. The results suggested a rigid and durable structure for CuFe_2_O_4_@SiO_2_@ZrO_2_/SO_4_^2−^/Cu as a heterogeneous nano-catalyst. Furthermore, to elucidate the chemical structure as well as stability of the catalyst, the catalyst recovered after the 9^th^ run was studied by FTIR, FE-SEM and TEM analyses (Fig. 6b–d). After comparing the FTIR spectrum of the recovered catalyst with the corresponding FTIR spectrum of the fresh catalyst, it was determined that the structure of the catalyst remained intact during the recycles (Fig. 6b). Moreover, the FE-SEM and TEM images of the recovered catalyst revealed that the morphology of the nanoparticles was the same as revealed in the corresponding images of the fresh catalyst (Fig. 6c and d), respectively. No agglomeration or increase in the particle size was observed even after nine consecutive recycles. Note that the catalyst did not show any detectable metal leaching even after the 9^th^ run. ICP analysis of the residue obtained from the mixture after the 9^th^ run was performed to separately investigate the presence of Fe, Cu, and Zr; for each experiment, a negligible amount of these elements was detected, which confirmed the heterogeneous nature as well as durability of the catalyst during the reactions (Table S1†).

The heterogeneous nature of the catalyst was studied by a hot filtration test.^42^ The aforementioned model reaction was applied for this test. The catalyst was magnetically removed after 10 min of the reaction (30% yield, GC analysis). The reaction was allowed to proceed, and the conversion was investigated after 2 h by GC. The reaction conversion reached 33%, which confirmed that CuFe_2_O_4_@SiO_2_@ZrO_2_/SO_4_^2−^/Cu operated heterogeneously in the mixture, and no metal leaching took place during the reaction.

We compared the catalytic activity of CuFe_2_O_4_@SiO_2_@ZrO_2_/SO_4_^2−^/Cu with those reported for the Sonogashira coupling reaction of phenyl acetylene with 4-Me-iodobenzene, 4-NO_2_-bromobenzene, and 4-MeO-iodobenzene. As shown in Table 4, the present methodology was superior to all the reported catalytic systems in terms of time, catalyst amount and yield of the reaction. Evidently, the reaction conditions were very mild, and the heterogeneous catalyst compromised some advantages such as easy preparation and recycling, minimum metal contamination and economic friendliness.

**Table 4:** Comparison of the catalytic activity of the CuFe_2_O_4_@SiO_2_@ZrO_2_/SO_4_^2−^/Cu NPs with examples from literature for the Sonogashira reaction between phenylacetylene and 4-Me-iodobenzene, 4-NO_2_-bromobenzene, and 4-MeO-iodobenzene

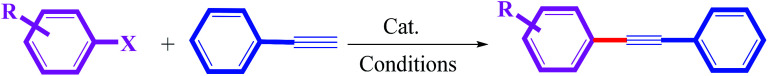							
Run	X	R	Catalyst	Condition	Time (h)	Yield (%)	Ref.
1	I	4-Me	PdCu@GQD@Fe_3_O_4_ (Pd 0.3 mol%, Cu 0.35 mol%)^a^	Toluene or DMA/DABCO/50 °C	24	91	1
2			Au·CuFe_2_O_4_@silica	DMA/*t*-BuOK/115 °C	48	96	2
3			MgO@PdCu (Pd 0.05 mol%, Cu 0.01 mol%)	DMF/DABCO/60 °C	24	97	43
4			CuI (0.2 mol%) PPh_3_ (4 mol%)	H_2_O/K_2_CO_3_/Ar/140 °C	24	93	11
5			Pd/Fe_3_O_4_ NPs (0.2 mol%)	DMF/piperidine/110 °C	24	83	44
6			CuFe_2_O_4_@SiO_2_@ZrO_2_/SO_4_^2−^/Cu (0.3 mol% Cu)	H_2_O/NaOH/60 °C	30 min	94	This work
7	Br	4-NO_2_	Pd-CS (0.1% mol)^b^	EtOH/H_2_O/K_2_CO_3_/65 °C	8	100	45
8			Pd/Fe_3_O_4_ NPs (0.2 mol%)	DMF/piperidine/110 °C	24	73	44
9			CuFe_2_O_4_@SiO_2_@ZrO_2_/SO_4_^2−^/Cu (0.3 mol% Cu)	H_2_O/NaOH/60 °C	45 min	97	This work
10	I	4-MeO	Fe_3_O_4_/AO/Pd (0.1 mol%)^c^	DMF/Et_3_N/80 °C	0.5	98	46
11			Pd/Fe_3_O_4_ NPs(0.2 mol%)	DMF/piperidine/110 °C	24	90	44
12			CuFe_2_O_4_@SiO_2_@ZrO_2_/SO_4_^2−^/Cu (0.3 mol% Cu)	H_2_O/NaOH/60 °C	55	88	This work

a GQD = graphene quantum dots.

b CS = chitosan.

c AO = amidoxime.

## 4. Conclusion

Herein, copper was incorporated into sulfated zirconium oxide (ZrO_2_/SO_4_^2−^/Cu) supported on copper ferrite nanoparticles (CuFe_2_O_4_ NPs); the resultant compound was found to be an efficient magnetically durable catalyst for the Sonogashira reaction in water. The catalyst demonstrated high efficiency not only for aryl iodides but also for aryl bromides and aryl chlorides. Note that the catalytic activity of the modified sulfated zirconium oxide in the organic synthesis has been rarely studied. The catalyst has a monoclinic-tetragonal mixed crystal structure, high thermal stability until 1000 °C, and a 10 emu g^−1^ saturation magnetization with a 40 nm average size and an irregular shape. The catalyst was further characterized by the EDX and FTIR analyses. This magnetic nanocatalyst could be recycled for at least 9 consecutive runs without any notable loss in activity. The study on the recovered catalyst revealed the high stability and durability of the proposed catalyst. The control experiments completely rule out the synergetic effect of ZrO_2_/SO_4_^2−^/Cu and CuFe_2_O_4_@SiO_2_, which leads to the incredible catalytic activity of the proposed catalyst; in literature, the interference of CuFe_2_O_4_@SiO_2_ in the Sonogashira reaction has also been demonstrated. An electron-transfer between Cu and Zr metal sites could be responsible for the proposed oxidative addition and reductive elimination mechanism, in agreement with literature. Furthermore, an interconversion between the sulfate ions on the catalyst surface mediated/facilitated the function of the base in water *via* adsorption of the cation. The current methodology can indeed replace the expensive Pd-based catalytic systems with highly toxic and expensive phosphine ligands to catalyze the Sonogashira cross-coupling reactions. The use of water as a solvent, short reaction time, high efficiency and absence of by-products are other advantages of the abovementioned catalyst.

## Conflicts of interest

There are no conflicts to declare.

## Supplementary Material

RA-009-C9RA03406D-s001
