# Supplementary material for: The CuFe2O4@SiO2@ZrO2/SO42−/Cu nanoparticles: an efficient magnetically recyclable multifunctional Lewis/Brønsted acid nanocatalyst for the ligand- and Pd-free Sonogashira cross-coupling reaction in water
Source: RSC Adv. 2019 Jul 3;9(36):20749–59. doi: 10.1039/c9ra03406d (PMC9065763; doi:10.1039/c9ra03406d)
Supplement: RA-009-C9RA03406D-s001 [file RA-009-C9RA03406D-s001.pdf]

Electronic Supplementary Material (ESI) for RSC Advances.
This journal is © The Royal Society of Chemistry 2019

Supplementary Materials

$CuFe_2O_4@SiO_2@ZrO_2/SO_4^{2-}/Cu$ nanoparticles: as an efficient magnetically recyclable multifunctional Lewis/Brønsted acid nanocatalyst for ligand- and Pd-free Sonogashira cross-coupling reaction in water

Mohammad Ali Nasseri,* Seyyedeh Ameneh Alavi, Milad Kazemnejadi, Ali Allahresani

Department of Chemistry, Faculty of Science, University of Birjand, P. O. Box 97175-615, Birjand, Iran.

* Corresponding author: manaseri@birjand.ac.ir (M. A. Nasseri)

Table S1 Metal leaching tests over the model reaction of iodobenzene with phenyl acetylene under optimized conditions for each of nine runs using a ICP-MS instrument[a,b]

Run	Leaching amount ($\mu g.L^{-1}$)		
	Cu	Fe	Zr
1	0.000	0	0
2	0.000	0	0
3	0.000	0	0
4	0.000	0	0
5	0.006	0	0
6	0.005	0	0
7	0.005	0	0
8	0.006	0	0
9	0.005	0	0

[a] The analyses were taken at 324.754 nm, 259.940 nm, and 343.823 nm for Cu, Fe, and Zr respectively.
[b] The leaching tests were also carried out with a ICP-OES instrument and didn't found any detectable metal in the residues

^{1}H NMR, ^{13}C NMR and Mass characterization data of the sonogashira products:

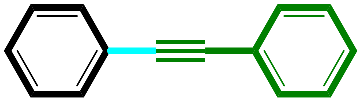

^{1}H-NMR (250 MHz, $CDCl_3$) δ: 7.23-7.28 (m, 6H), 7.43-7.47 (m, 4H) ppm; ^{13}C-NMR (62.9 MHz, $CDCl_3$) δ: 89.5, 123.3, 128.3, 129.2, 131.6 ppm; MS (m/e) = 178 [M^+]; Elemental Analysis: Calcd. C: 94.33, H: 5.67%, Found. C: 94.11, H: 5.89%.

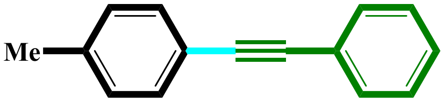

^{1}H-NMR ($CDCl_3$, 250 MHz) δ: 2.22 (s, 3H, CH_3), 7.14 (d, 2H, J= 8.4 Hz, Ar-H), 7.19-7.42 (m, 7H, Ar-H) ppm; ^{13}C-NMR (62.9 MHz, $CDCl_3$) δ: 21.5, 88.7, 89.6, 120.2, 123.5, 128.1, 128.3, 129.1, 131.5, 131.7, 138.4 ppm; MS (m/e)= 192 [M^+]; Elemental Analysis: Calcd. C: 93.77, H: 6.30%, Found. C: 93.57, H: 6.23%.

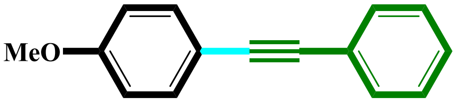

^{1}H-NMR (250 MHz, $CDCl_3$) δ: 3.76 (s, 3H, CH_3), 6.79 (d, 2H, J= 8.2 Hz, Ar-H), 7.21-7.25 (m, 3H, Ar-H), 7.37-7.44 (m, 4H, Ar-H) ppm; ^{13}C-NMR (62.9 MHz, $CDCl_3$) δ: 55.2, 88.0, 89.4, 114.0, 115.3, 123.6, 127.9, 128.3, 131.4, 133.0, 159.6 ppm; MS (m/e)= 208 [M^+]; Elemental Analysis: Calcd. C: 86.50, H: 5.82%, Found. C: 86.64, H: 5.71%.

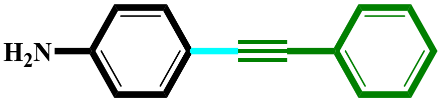

^{1}H-NMR (250 MHz, $CDCl_3$) δ: 3.62 (s, 2H, NH_2), 6.52 (s, 2H, Ar-H), 7.22-7.40 (m, 7H, Ar-H) ppm; ^{13}C-NMR (62.9 MHz, $CDCl_3$) δ: 87.3, 90.2, 112.5, 114.7, 123.9, 127.7, 128.3, 131.3, 132.9, 146.7 ppm.

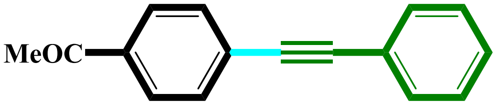

^{1}H-NMR (250 MHz, $CDCl_3$) δ: 2.51 (s, 3H, CH_3), 7.16-7.33 (m, 3H, Ar-H), 7.45-7.54 (m, 4H, Ar-H), 7.86 (d, 2H, *J*=7.5 Hz, Ar-H) ppm; ^{13}C-NMR (62.9 MHz, $CDCl_3$) δ: 27.0, 88.9, 92.9, 123.0, 128.2, 128.3, 128.8, 129.5, 132.0, 132.2, 136.5, 197.6 ppm.

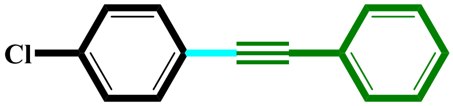

^{1}H NMR (250 MHz, $CDCl_3$) δ: 7.13-7.43 (m, 9H, Ar-H) ppm; ^{13}C NMR (62.9 MHz, $CDCl_3$) δ: 88.2, 90.3, 121.8, 122.9, 128.4, 128.5, 128.7, 131.6, 132.8, 134.2 ppm.

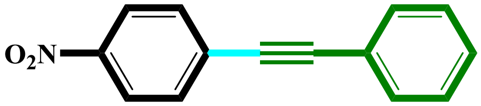

^{1}H-NMR (250 MHz, $CDCl_3$) δ: 7.37-7.41 (m, 3H, Ar-H), 7.54-7.57 (m, 2H, Ar-H), 7.68 (d, 2H, *J*= 12.5 Hz, Ar-H), 8.22 (d, 2H, *J*= 7.5 Hz, Ar-H) ppm; ^{13}C-NMR (62.9 MHz, $CDCl_3$) δ: 87.5, 94.7, 122.0, 123.6, 128.5, 129.2, 130.2, 131.8, 132.2, 146.9 ppm; MS (m/e)= 224 [M^+]; Elemental Analysis: Calcd. C: 75.32, H: 4.07, N: 6.27%, Found. C: 75.42, H: 4.21, N: 6.15%.

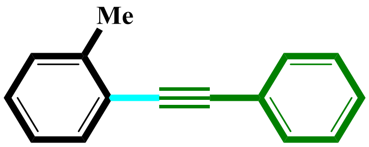

^{1}H NMR (250 MHz, $CDCl_3$) δ: 2.44 (s, 3H, CH_3), 7.14-7.46 (m, 9H, Ar-H) ppm; ^{13}C NMR ($CDCl_3$, 62.9 MHz) δ: 20.7, 86.01, 94.2, 123.0, 125.5, 128.1, 128.30, 128.35, 129.4, 131.5, 131.8, 140.1 ppm; MS (m/e)= 192 [M^+]; Elemental Analysis: Calcd. C: 93.70, H: 6.30%, Found. C: 93.82, H: 6.18%.

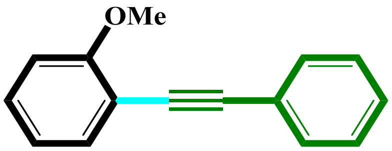

^{1}H NMR (250 MHz, $CDCl_3$) δ: 3.82 (s, 3H, CH_3), 6.87 (d, 2H, J= 7.25 Hz, Ar-H), 7.31-7.33 (m, 3H, Ar-H), 7.45-7.53 (m, 4H, Ar-H) ppm; ^{13}C NMR ($CDCl_3$, 62.9 MHz): δ: 55.8, 85.6, 93.3, 110.6, 112.4, 120.4, 123.5, 128.0, 128.1, 129.7, 131.6, 133.5, 159.8 ppm.

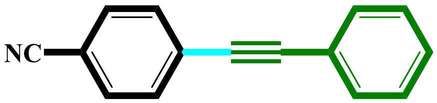

^{1}H-NMR (250 MHz, $CDCl_3$) δ: 7.28-7.31 (m, 3H, Ar-H), 7.44-7.53 (m, 6H, Ar-H) ppm; ^{13}C-NMR (62.9 MHz, $CDCl_3$) δ: 87.7, 93.7, 111.4, 118.5, 122.2, 128.2, 128.5, 129.1, 131.7, 132.03, 132.06 ppm; MS (m/e)= 203 [M^+]; Elemental Analysis: Calcd. C: 88.64, H: 4.47, N: 6.89%, Found. C: 88.77, H: 4.57, N: 6.66%.

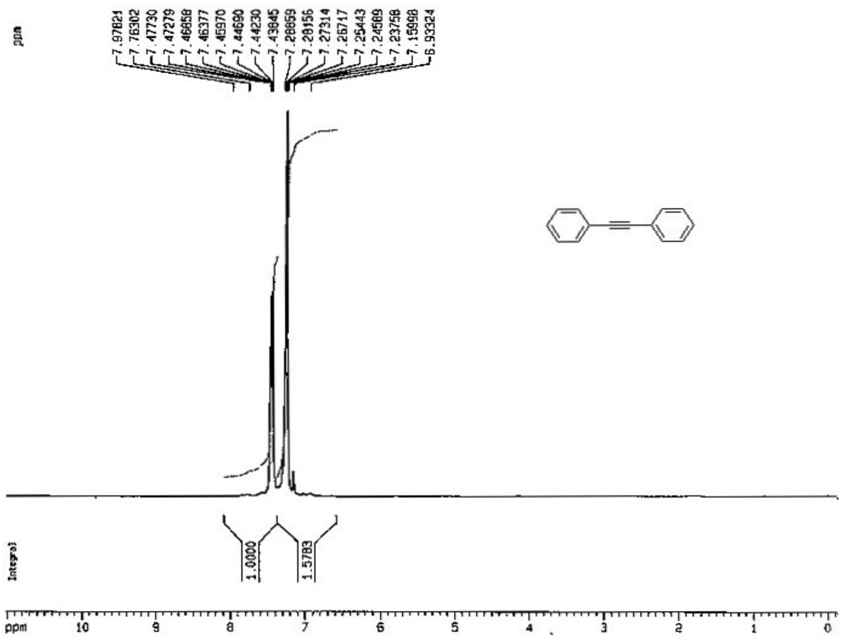
ppm
7.97821
7.76302
7.47730
7.47279
7.46858
7.46377
7.45970
7.44690
7.44230
7.43845
7.28869
7.28156
7.27314
7.26717
7.25443
7.24589
7.23758
7.15998
6.93324
Integral
1.0000
1.5783
ppm 10 9 8 7 6 5 4 3 2 1 0

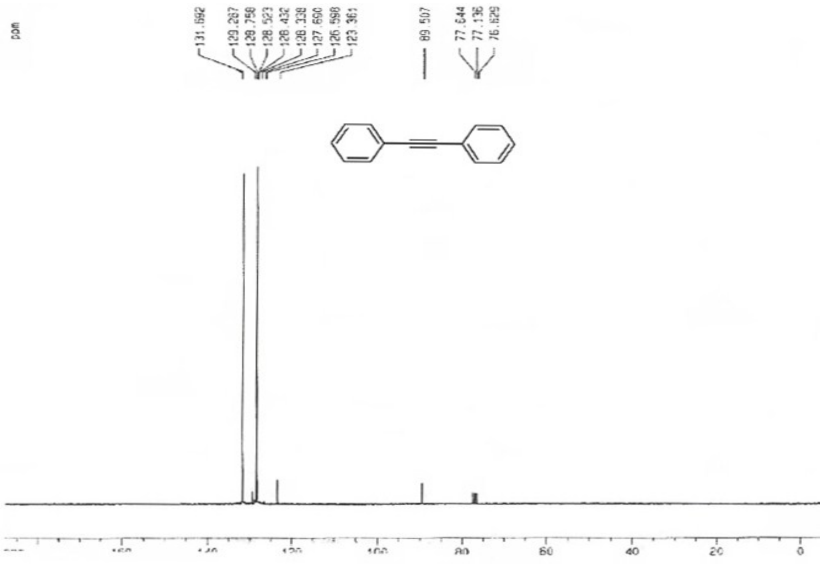
ppm
131.692
129.287
128.758
128.523
128.432
128.338
127.690
126.598
123.361
89.507
77.644
77.136
76.629
80 60 40 20 0

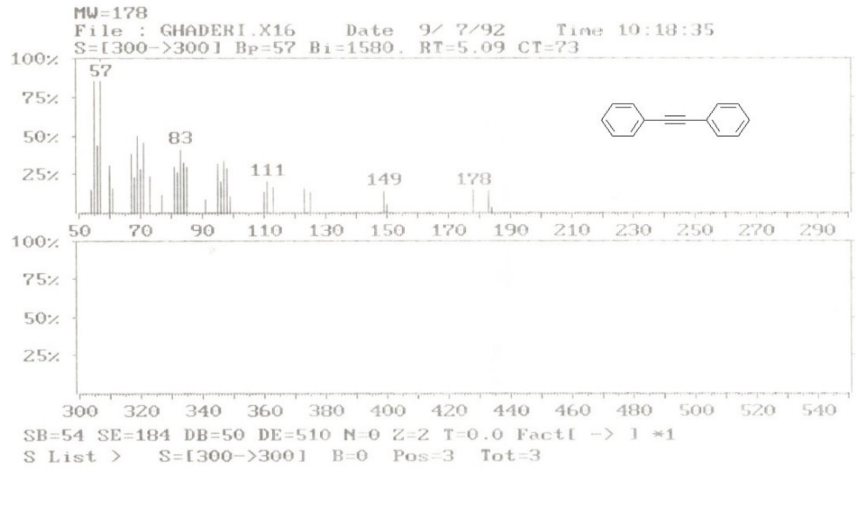
MW=178
File : GHADERI.X16 Date 9/ 7/92 Time 10:18:35
S=[300->300] Bp=57 Bi=1580. RT=5.09 CT=73
100% 75% 50% 25%
57
83
111
149
178
50 70 90 110 130 150 170 190 210 230 250 270 290
300 320 340 360 380 400 420 440 460 480 500 520 540
SB=54 SE=184 DB=50 DE=510 N=0 Z=2 T=0.0 Fact[->] *1
S List > S=[300->300] B=0 Pos=3 Tot=3

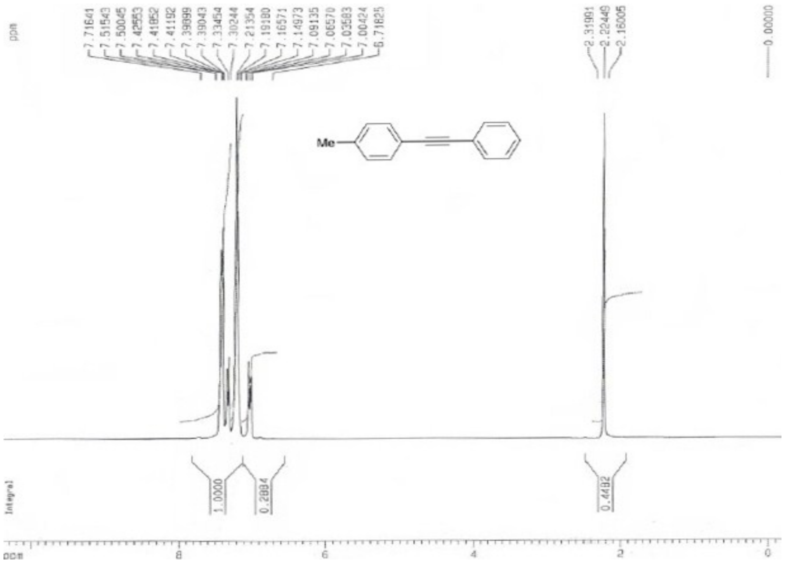
ppm
7.71641
7.51543
7.50045
7.42553
7.41852
7.41192
7.39899
7.39043
7.33454
7.30244
7.21354
7.19180
7.16571
7.14973
7.09135
7.06570
7.03583
7.00424
6.71625
2.31991
2.22449
2.16005
0.00000
Me
Integral
1.0000
0.2884
0.4482
ppm
8
6
4
2
0

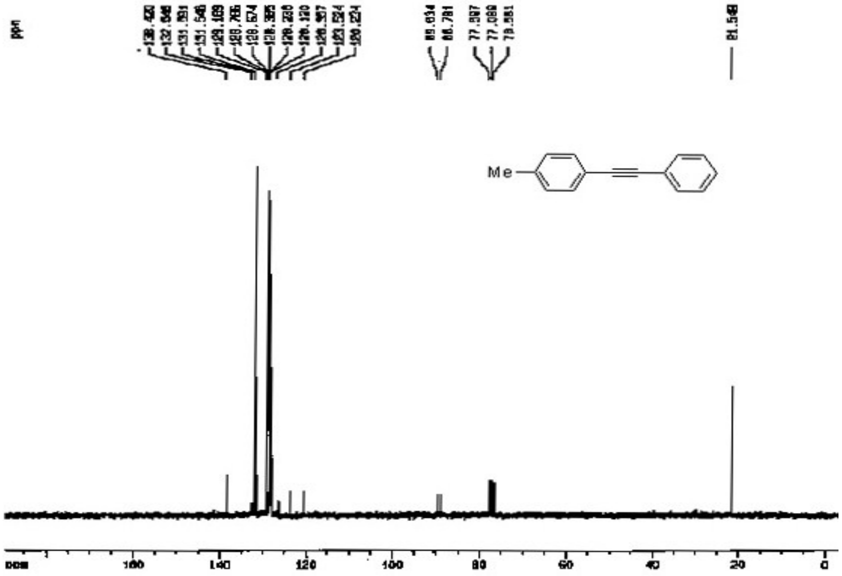
ppm
Me
ppm
140
120
100
80
60
40
20
0

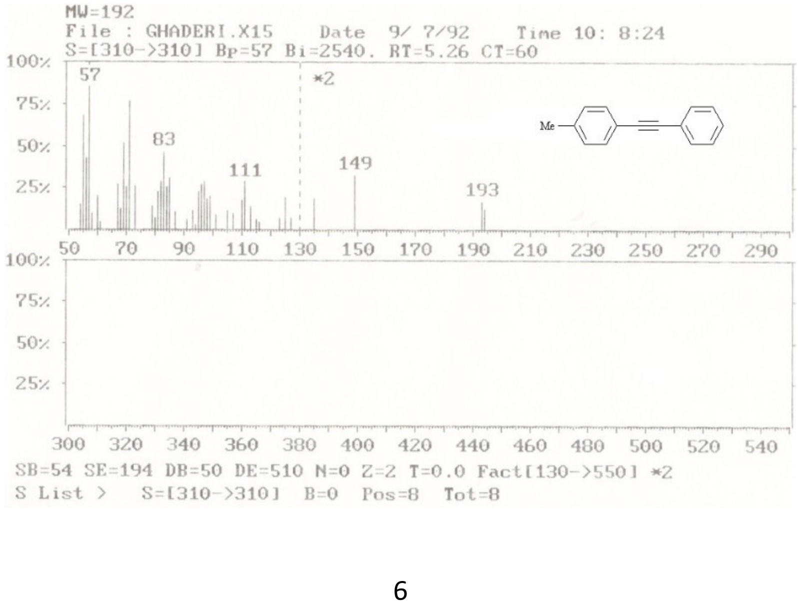
MW=192
File : GHADERI.X15 Date 9/ 7/92 Time 10: 8:24
S=[310->310] Bp=57 Bi=2540. RT=5.26 CT=60
100%
75%
50%
25%
57
83
111
149
193
*2
Me
50 70 90 110 130 150 170 190 210 230 250 270 290
100%
75%
50%
25%
300 320 340 360 380 400 420 440 460 480 500 520 540
SB=54 SE=194 DB=50 DE=510 N=0 Z=2 T=0.0 Fact[130->550] *2
S List > S=[310->310] B=0 Pos=8 Tot=8

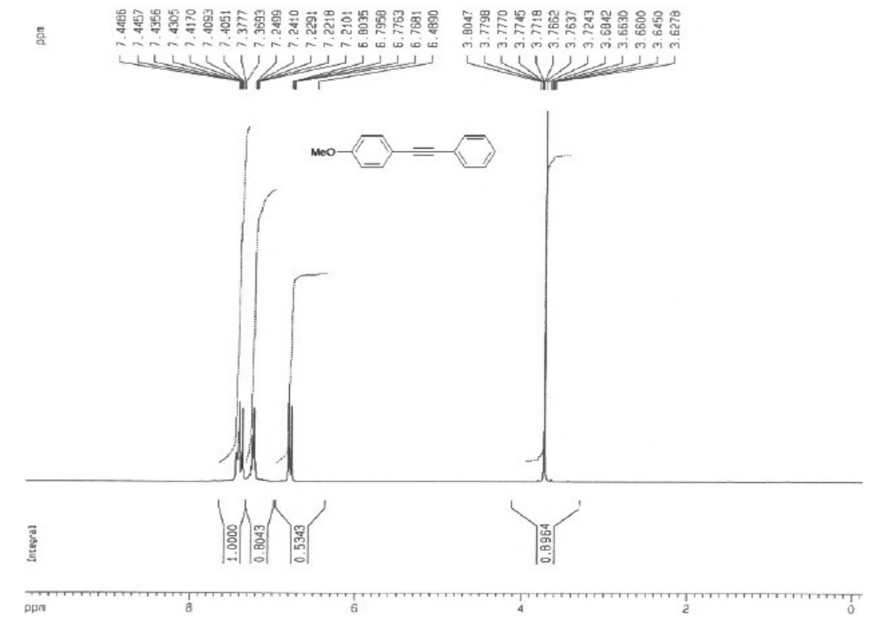
ppm
7.4486 7.4457 7.4356 7.4305 7.4170 7.4093 7.4051 7.3777 7.3693 7.2499 7.2410 7.2291 7.2218 7.2101 6.8035 6.7958 6.7763 6.7681 6.4890
3.8047 3.7798 3.7770 3.7745 3.7718 3.7662 3.7637 3.7243 3.6842 3.6630 3.6600 3.6450 3.6278
MeO
Integral
1.0000 0.8043 0.5343 0.8964
ppm 8 6 4 2 0

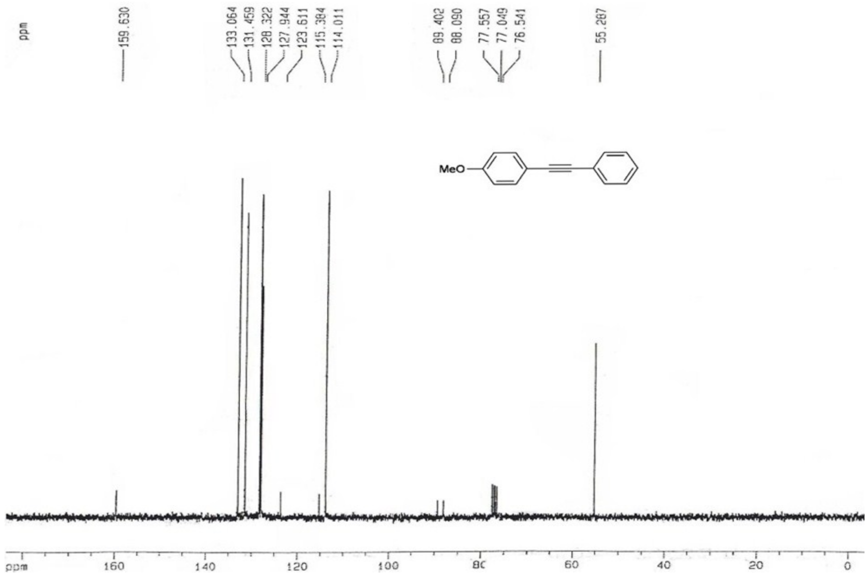
ppm
159.630
133.064 131.459 128.322 127.944 123.611 115.384 114.011
89.402 88.090 77.557 77.049 76.541
55.287
MeO
ppm 160 140 120 100 80 60 40 20 0

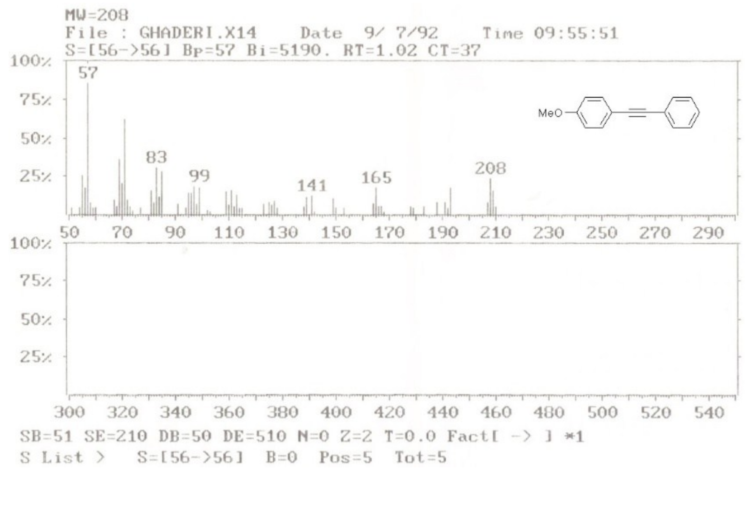
MW=208
File : GHADERI.X14 Date 9/ 7/92 Time 09:55:51
S=[56->56] Bp=57 Bi=5190. RT=1.02 CT=37
100% 75% 50% 25%
57 83 99 141 165 208
MeO
50 70 90 110 130 150 170 190 210 230 250 270 290
300 320 340 360 380 400 420 440 460 480 500 520 540
SB=51 SE=210 DB=50 DE=510 N=0 Z=2 T=0.0 Fact[->] *1
S List > S=[56->56] B=0 Pos=5 Tot=5

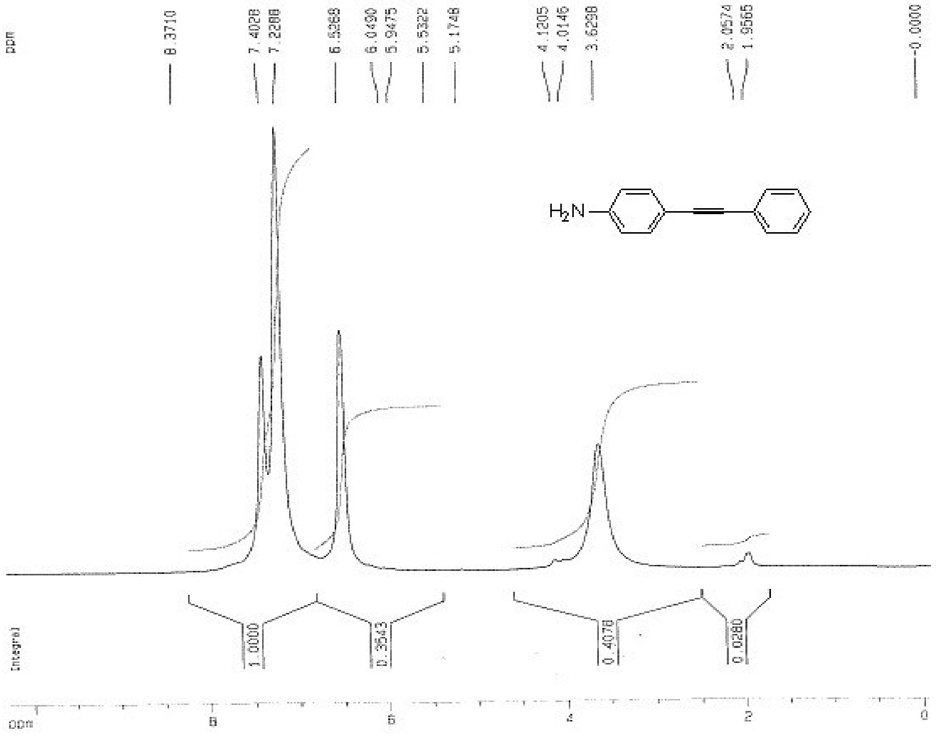

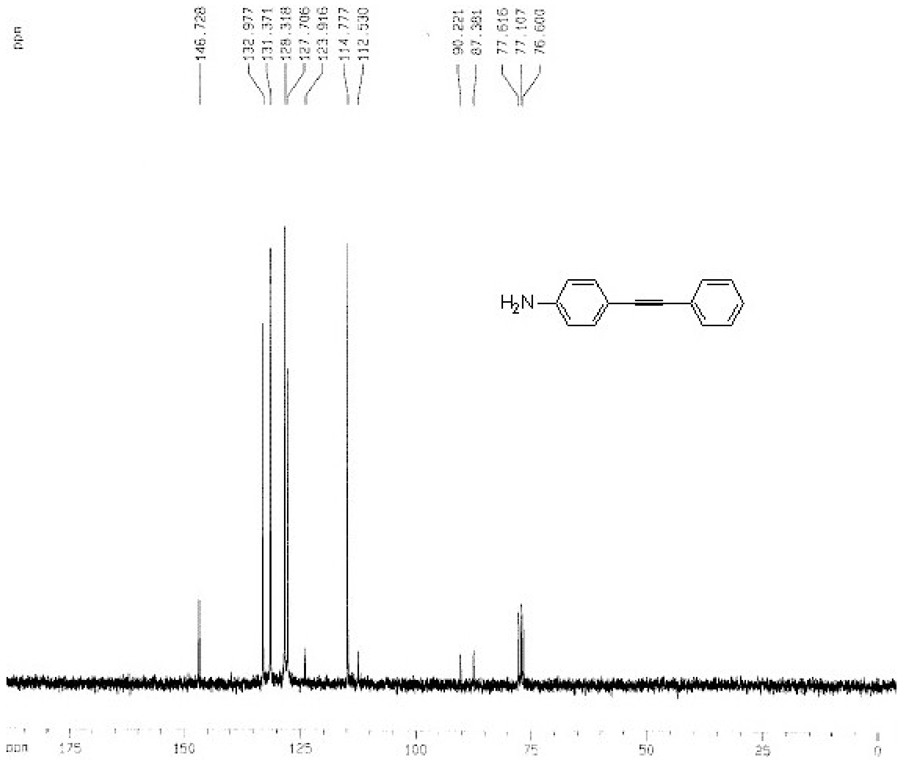

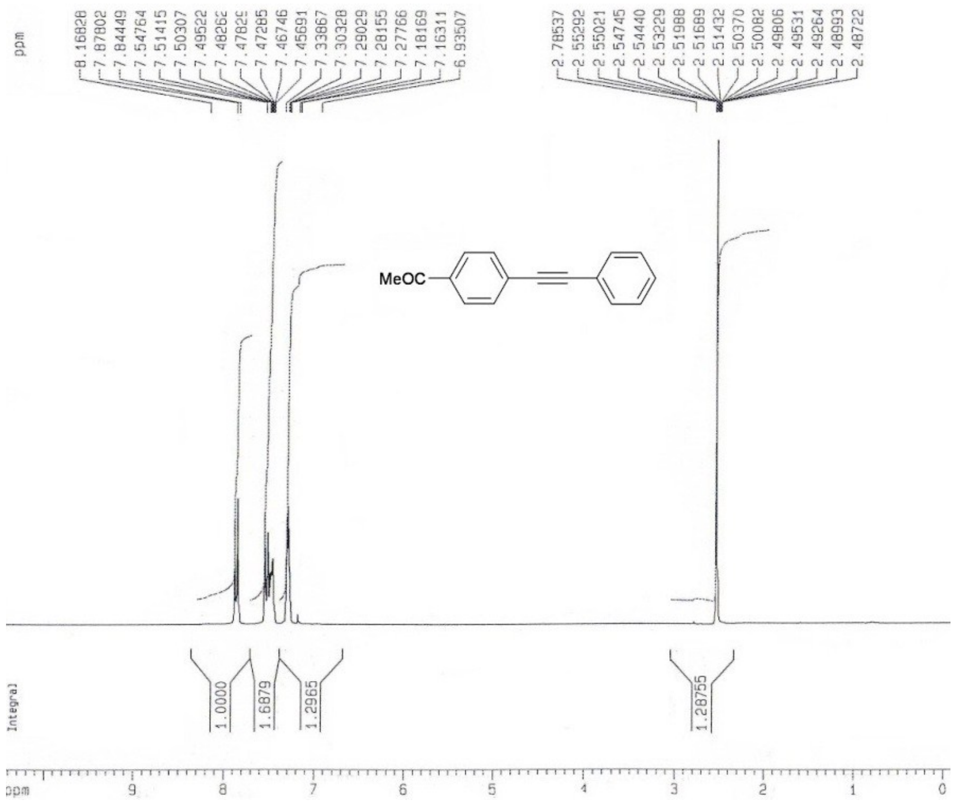
ppm
8.16828
7.87802
7.84449
7.54764
7.51415
7.50307
7.49522
7.48262
7.47829
7.47285
7.46746
7.45691
7.33867
7.30328
7.29029
7.28155
7.27766
7.18169
7.16311
6.93507
2.78537
2.55292
2.55021
2.54745
2.54440
2.53229
2.51988
2.51689
2.51432
2.50370
2.50082
2.49806
2.49531
2.49264
2.48993
2.48722
MeOC
Integral
1.0000
1.6879
1.2965
1.28755
ppm
9
8
7
6
5
4
3
2
1
0

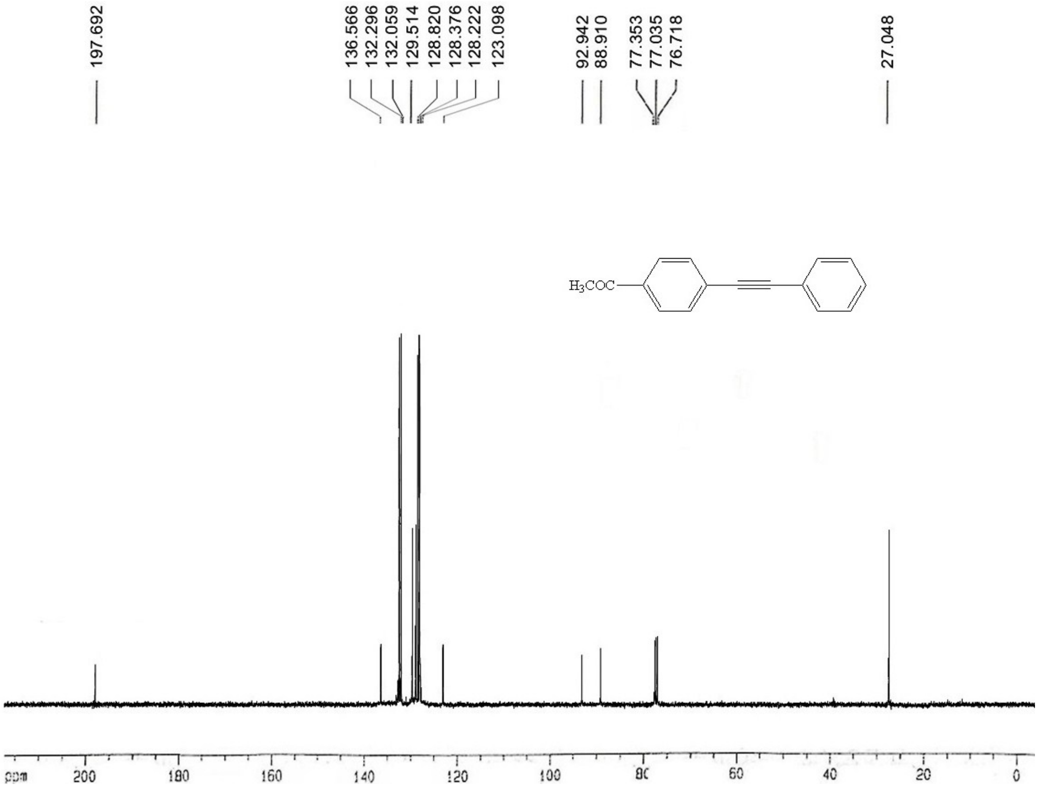
197.692
136.566
132.296
132.059
129.514
128.820
128.376
128.222
123.098
92.942
88.910
77.353
77.035
76.718
27.048
H3COC
ppm
200
180
160
140
120
100
80
60
40
20
0

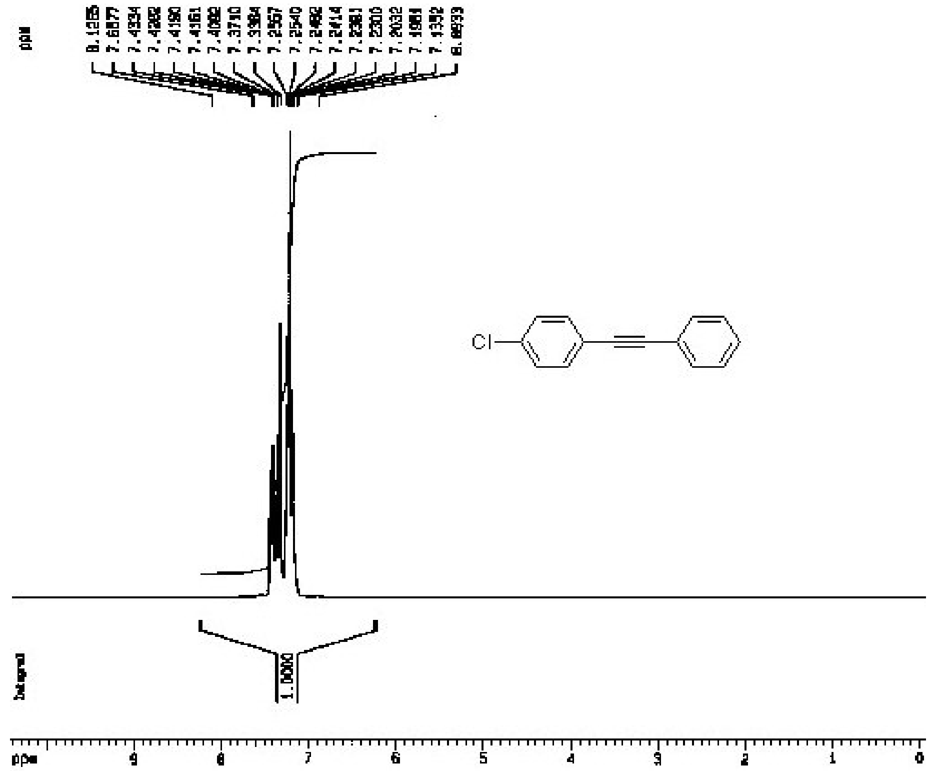

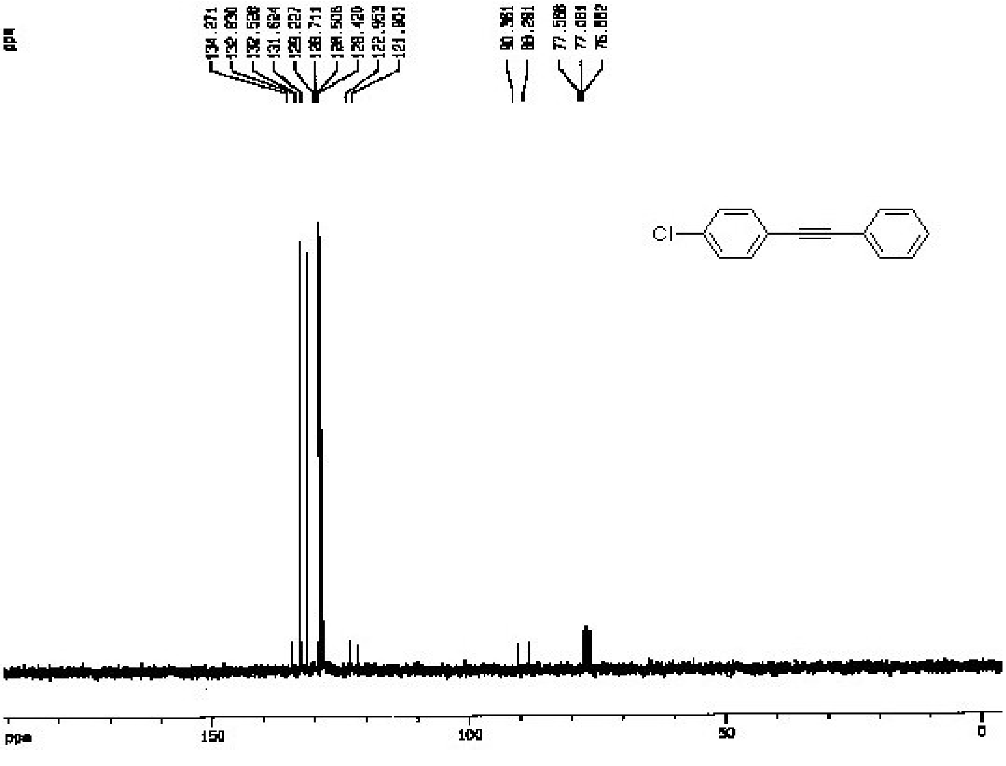

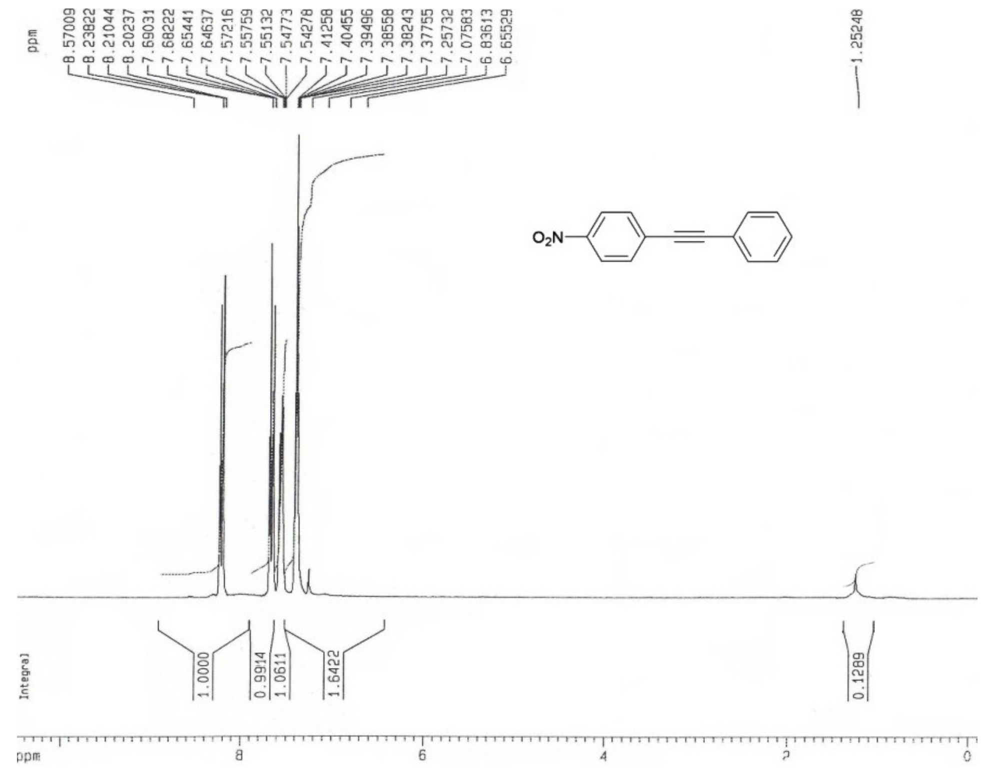
ppm
8.57009
8.23822
8.21044
8.20237
7.69031
7.68222
7.65441
7.64637
7.57216
7.55759
7.55132
7.54773
7.54278
7.41258
7.40455
7.39496
7.38558
7.38243
7.37755
7.25732
7.07583
6.83613
6.65529
1.25248
O2N
Integral
1.0000
0.9914
1.0611
1.6422
0.1289
ppm
8
6
4
2
0

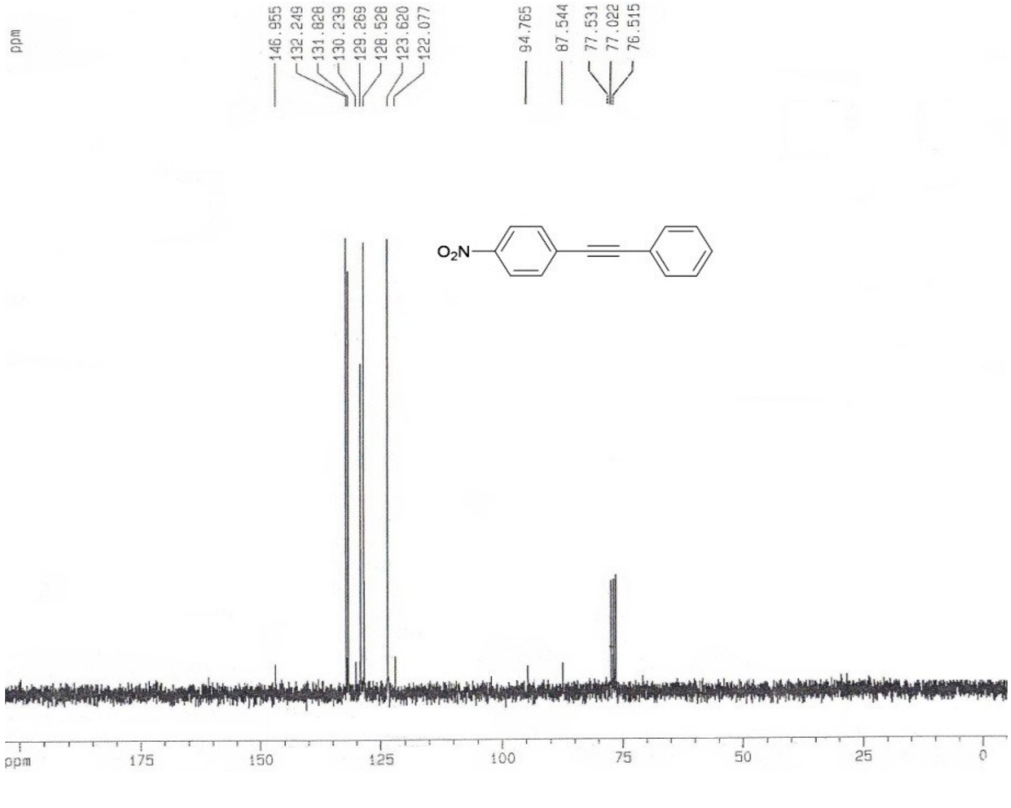
ppm
146.955
132.249
131.828
130.239
129.269
128.528
123.620
122.077
94.765
87.544
77.531
77.022
76.515
O2N
ppm
175
150
125
100
75
50
25
0

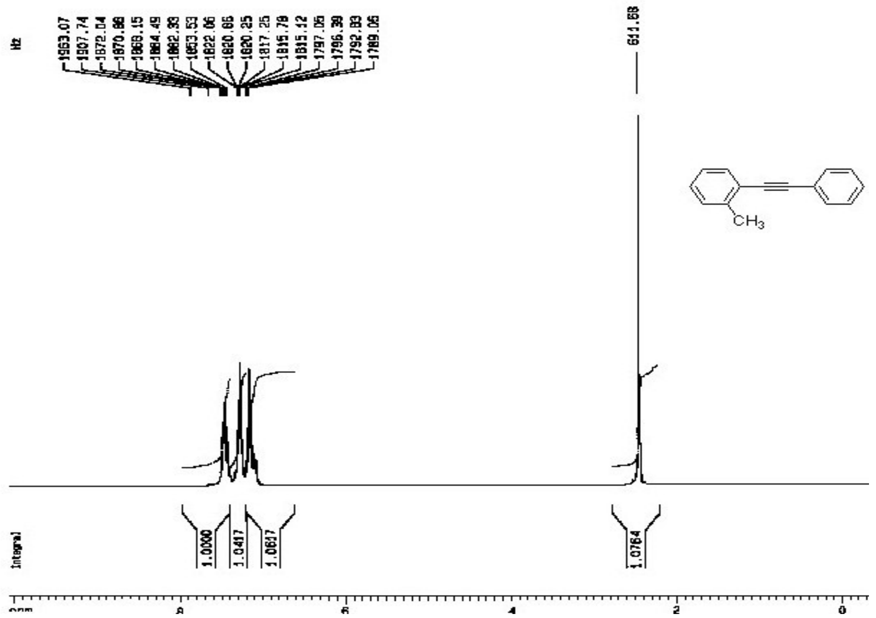
Hz
1963.07
1907.74
1872.04
1870.86
1868.15
1864.49
1862.33
1853.53
1822.06
1820.65
1820.25
1817.25
1815.78
1815.12
1797.05
1796.39
1792.83
1789.05
611.68
CH3
Integral
1.0000
1.0417
1.0697
1.0764
ppm

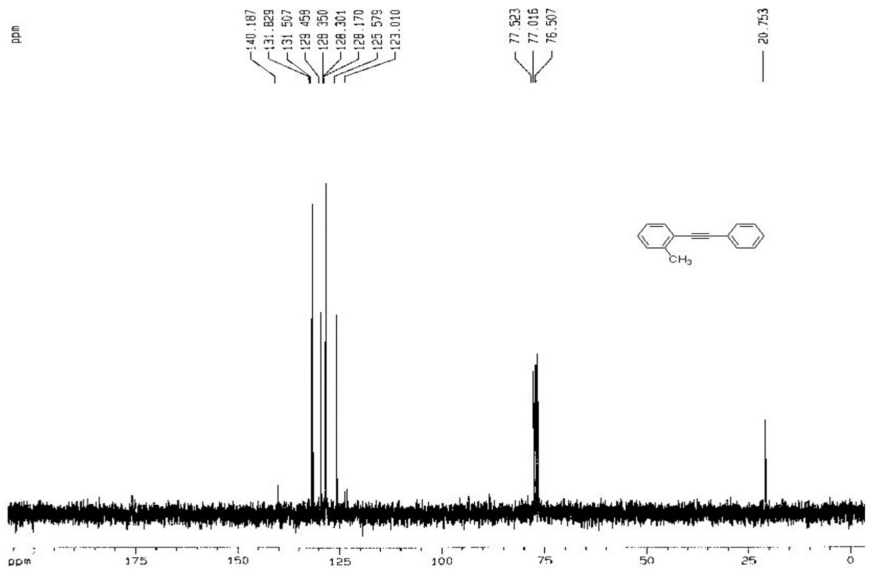
ppm
140.187
131.829
131.507
129.458
128.350
128.301
128.170
125.579
123.010
77.523
77.016
76.507
20.753
CH3
ppm
175
150
125
100
75
50
25
0

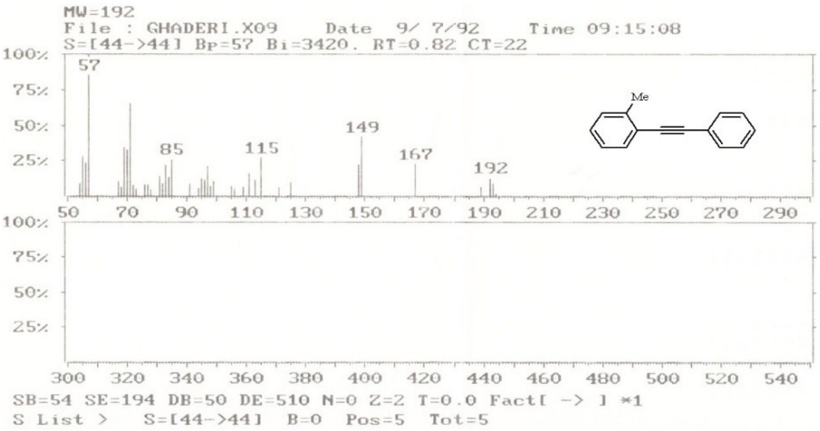
MW=192
File : GHADERI.X09 Date 9/ 7/92 Time 09:15:08
S=[44->44] Bp=57 Bi=3420. RT=0.82 CT=22
57
85
115
149
167
192
Me
SB=54 SE=194 DB=50 DE=510 N=0 Z=2 T=0.0 Fact[->] *1
S List > S=[44->44] B=0 Pos=5 Tot=5

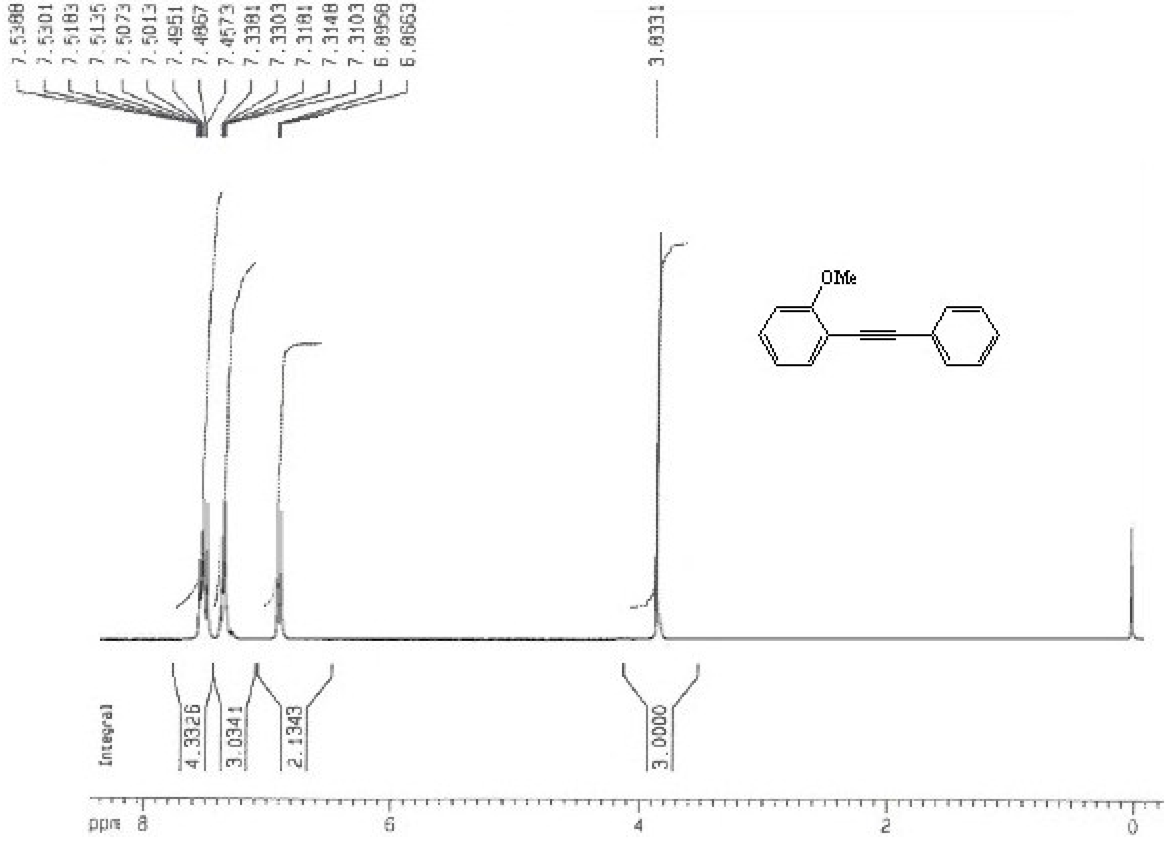
7.5300
7.5301
7.5183
7.5135
7.5073
7.5013
7.4951
7.4867
7.4573
7.3381
7.3303
7.3181
7.3140
7.3103
6.8950
6.8663
3.8331
OMe
Integral
4.3326
3.0341
2.1343
3.0000
ppm

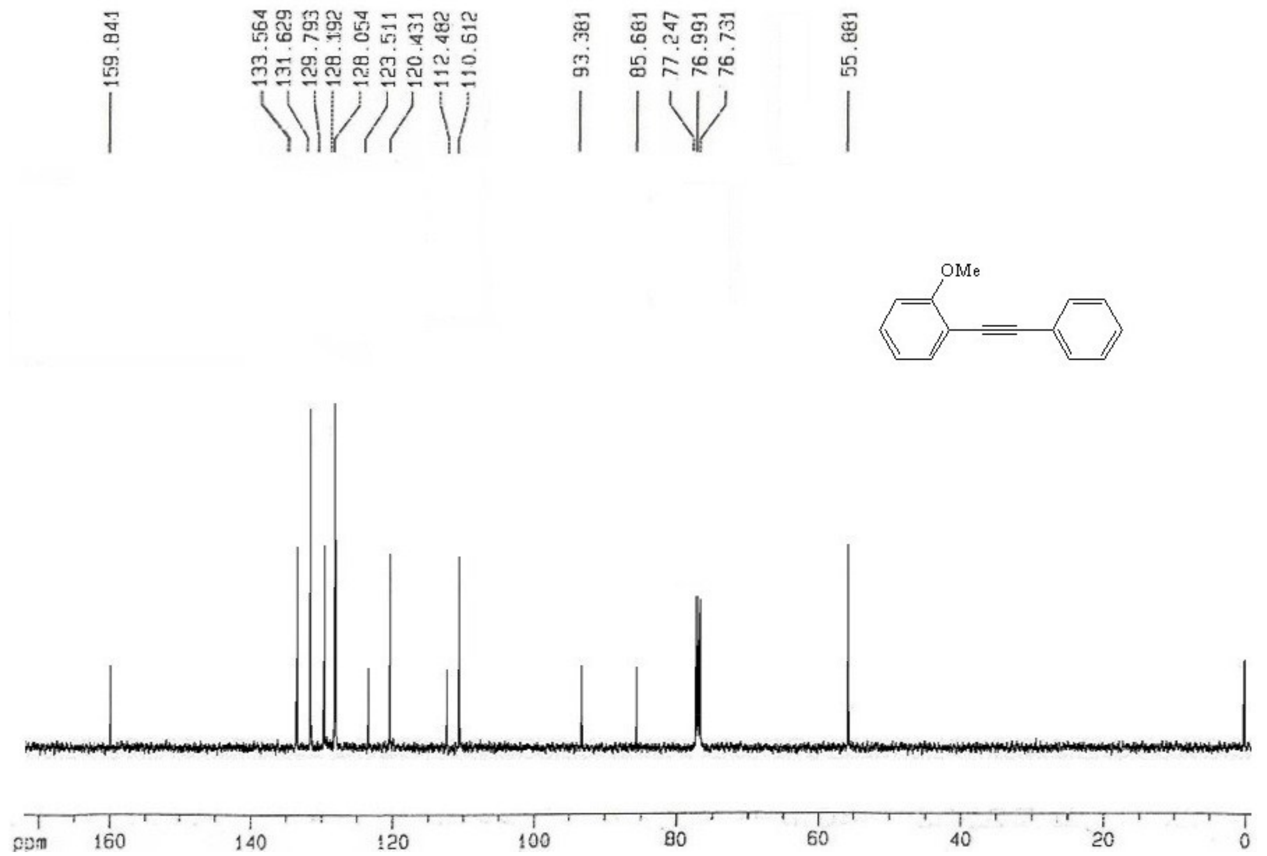

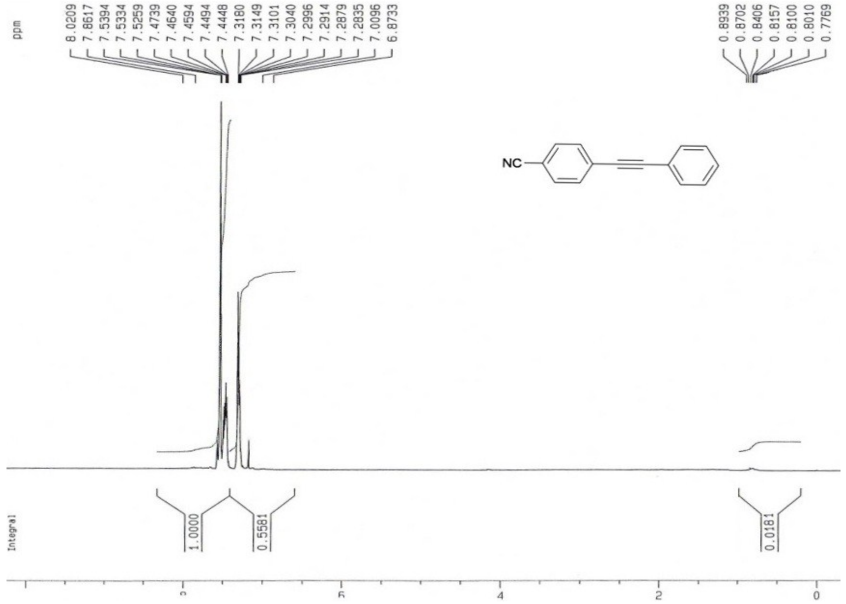

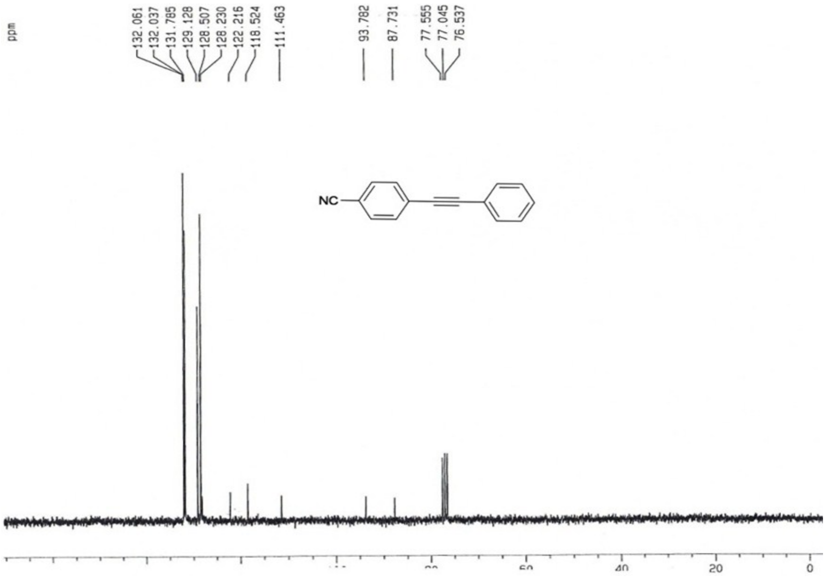
ppm
132.061
132.037
131.785
129.128
128.507
128.230
122.216
118.524
111.463
93.782
87.731
77.555
77.045
76.537
NC
60
40
20
0

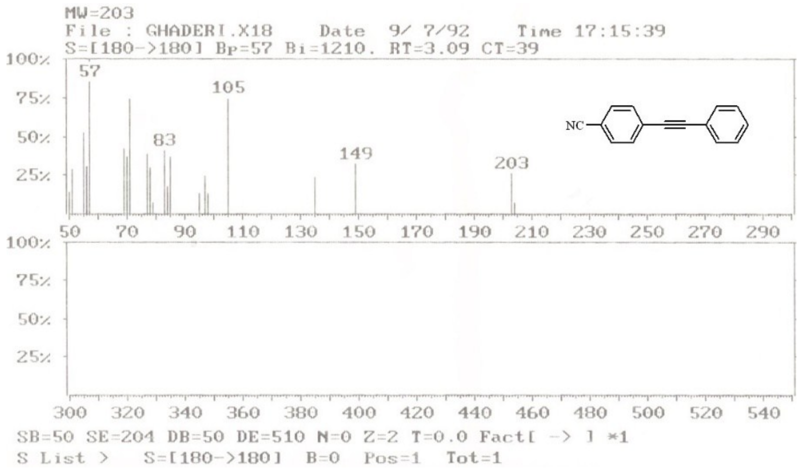
MW=203
File : GHADERI.X18 Date 9/ 7/92 Time 17:15:39
S=[180->180] Bp=57 Bi=1210. RT=3.09 CT=39
57
105
83
149
203
NC
100%
75%
50%
25%
50 70 90 110 130 150 170 190 210 230 250 270 290
300 320 340 360 380 400 420 440 460 480 500 520 540
SB=50 SE=204 DB=50 DE=510 N=0 Z=2 T=0.0 Fact[->] *1
S List > S=[180->180] B=0 Pos=1 Tot=1
